# Supplementary material for: Study protocol for a multicentre, randomised, controlled trial to assess the effectiveness of antimicrobial central venous catheters versus ordinary central venous catheters at reducing catheter related infections in critically ill Chinese patients
Source: BMJ Open. 2017 Dec 29;7(12):e016564. doi: 10.1136/bmjopen-2017-016564 (PMC5778275; doi:10.1136/bmjopen-2017-016564)
Supplement: Supplementary file 1 [file bmjopen-2017-016564supp001.pdf]

Supplemental Appendix

This appendix has been provided by authors to give readers additional information about the research.

Study protocol for a multi-centre, randomized, controlled trial to assess the effectiveness of antimicrobial central venous catheters versus ordinary central venous catheters at reducing catheter-related infections in critically ill Chinese patients

Minming Wu[1], Yao Chen[1], Du Bin[2], Kang Yan[1]

[1] Department of Critical Care Medicine, West China Hospital of Medicine, Sichuan University, No. 37 Guo Xue Xiang, Chengdu, Sichuan, 610041, P.R. China

[2] Medical Intensive Care Unit, Peking Union Medical College Hospital, Peking Union Medical College and Chinese Academy of Medical Sciences, Beijing 100730, China

Corresponding author

Dr Kang Yan

Intensive Care Unit, West China Hospital of Medicine, Sichuan University, Chengdu, Sichuan, 610041, China

Tel.: +86 18980601566

Fax: 85422508

Email: kang_yan_123@163.com

Table of contents

1) Supplemental Background – Study catheter

2) Supplemental Method

  2.1) Procedures for insertion

  2.2) Care of the catheter during indwelling catheterization

  2.3) Remove catheter

  2.4) Indication of removal

  2.5) Test

  2.6) Analysis set

3) Supplemental tables

4）Supplementary Appendix References

1) **Study catheter**

Intervention catheters are antimicrobially modified. Polarization of the catheter material destroys the cell wall structure of microorganisms in case of surface colonization. Perpetual chemical interaction between the polyurethane of the catheter and the agent biguanide ensures the reduction of catheter-related infections during the entire application of the catheter. Control catheters are standard common dual-lumen catheters. The two kinds of catheters are distinguishable in appearance and packaging.

The new antimicrobial CVC (Certofix® protect) was developed by B.Braun to reduce the risk of CRI and CRT. A prospective, randomized, double-bind clinic trial (NCT00555282) conducted in the Czech Republic found that the rate of blood stream infection (BSI) was significantly lower in protected CVCs (2.00 % vs. 6.47 %, p=0.008), and the incidence of BSI/ 1000 catheter-days was lower in coated catheters (3.21 vs. 8.30, p=0.036) as well, but the coated CVC displayed similar incidence of the standard CVCs (17.36 % vs. 18.67 %, p=0.747) as well as incidence of catheter-related BSI (1.33 % vs. 1.94 %, p=0.752) [1].

2) **Supplemental Methods**

Training before trail [2, 3, 4, 5]

2.1) Procedures for insertion

First doctors chose a proper insertion site, and then used maximal barrier precautions during insertion (the operator was required to wear masks, sterile gloves, and surgical gowns and use large sterile drapes). After disinfected with povidone iodine or chlorhexidine, the catheter was inserted percutaneously using Seldinger technique. It was not allowed to exchange the catheter over a guidewire into an old site. Sites were dressed with hyalo-dressing.

2.2) Care of the catheter during indwelling catheterization

Twice a week or according to routine procedures, perform the follows: the dressing removed; the site inspected and cleaned with povidone-iodine or chlorhexidine; and the new dressing applied.

2.3) Remove catheters

At removal, the site was again disinfected by povidone iodine or chlorhexidine to make sure that the skin around the catheter was clean.

2.4) Indication of removal

No need for CVC in patients; Occlusion of catheter; Suspected or confirmed deep vein thrombosis of insertion site; Patients with highly suspected CRBSI and meeting one of the following criteria, haemodynamic instability, bacteremia; or the doctor in charge insisting to remove the catheter after 5 days' observation.

2.5) Tests

Blood culture

For the dual-lumen catheter, blood samples were taken from both lumens separately. Researchers should insert percutaneously to take sample from peripheral blood vessels. Aerobic culture and anaerobic culture were needed for each blood sample.

Cultures of CVC-tip

The entire catheter was removed aseptically, and 4-cm segment was cut from the catheter tip, which was semi-quantitatively cultured using the roll-plate method.

Vein ultrasound

It is used to diagnose or to exclude deep vein thrombosis (DVT). If the insertion site is femoral vein, doctors will screen iliac vein and femoral vein on both sides for DVT. While in the jugular vein, bilateral jugular veins should be inspected. Ultrasound is needless only in case of catheterization in subclavian vein. Ultrasound will be arranged before insertion and after withdrawal of catheter (within 48h).

2.6) Analysis set

Full analysis set (FAS)

The basic intention-to-treat (ITT) principle is that participants in the trials should be analyzed in the groups to which they are randomized, regardless of whether they receive or adhere to the allocated intervention. Based on ITT principle, FAS represents remaining participants after eliminating the least number of patients with reasonable way, including all the patients who are randomized and receive study catheters.

Per protocol set (PPS)

PPS can only be restricted to the participants who fulfill the protocol in the terms of the eligibility, interventions, and outcome assessment. Also, the PPS restricts the comparison of the treatments to the ideal patients, that is, those who adhere perfectly to the clinical trial instructions as stipulated in the protocol. [6]

3) **Supplemental tables**

Table 1 Time to visit and data collection

	Enrollment	Allocation	Post allocation	Closeout
Informed consent	×			
Inclusion/exclusion criteria	×			
Randomization	×			
Medical history & physical examination	×			
Temperature		×	×	
Insertion		×		
Blood test		×	×	
Blood culture			×	
Culture of CVC			×	
Vein ultrasound		×	×	
AE/SAE	×	×	×	×
Treatment/drug combination	×	×	×	×

Table 2 Alpha spending function and cut off value

	Lower bound	Upper bound	Alpha size of test	Alpha spending	Cumulative alpha	Power of test	Overall efficiency
Interim analysis	-2.96259	2.96259	0.003051	0.003051	0.003051	0.164276	0.164276
Final analysis	-1.96857	1.96857	0.049002	0.046949	0.050000	0.636018	0.800294

The distribution of suspension boundary (alpha) is normal distribution.

4) **Supplementary Appendix References**

1. http://braunoviny.bbraun.cz/clanky/polyhexanide-anti-infective-coating-ofcentral-venous/

2. http://www.safeinfusiontherapy.com/documents/french/Certofix_Brochure.pdf

3. 中华医学会重症医学分会。血管内导管相关感染的预防与治疗指南。中国实用外科杂志 2008; 28: 413-21

4. McGee DC, Gould MK. Preventing complications of central venous catheterization. N Engl J Med 2003;348(12):1123-33.

5. O'Grady N, Alexander M, Burns LA, et al. Guidelines for the prevention of intravascular catheter-related infections, 2011. Am J Infect Control 2011; 39(4 Suppl 1): S1-34

6. U.S. Department of Health and Human Services. Guidance for Industry E9, Statistical Principles for Clinical Trials. 1998.
