# Supplementary material for: Study protocol for a multicentre, randomised, controlled trial to assess the effectiveness of antimicrobial central venous catheters versus ordinary central venous catheters at reducing catheter related infections in critically ill Chinese patients
Source: BMJ Open. 2017 Dec 29;7(12):e016564. doi: 10.1136/bmjopen-2017-016564 (PMC5778275; doi:10.1136/bmjopen-2017-016564)
Supplement: Author's manuscript [file bmjopen-2017-016564.draft_revisions.pdf]

BMJ Open is committed to open peer review. As part of this commitment we make the peer review history of every article we publish publicly available.

When an article is published we post the peer reviewers' comments and the authors' responses online. We also post the versions of the paper that were used during peer review. These are the versions that the peer review comments apply to.

The versions of the paper that follow are the versions that were submitted during the peer review process. They are not the versions of record or the final published versions. They should not be cited or distributed as the published version of this manuscript.

BMJ Open is an open access journal and the full, final, typeset and author-corrected version of record of the manuscript is available on our site with no access controls, subscription charges or pay-per-view fees (http://bmjopen.bmj.com).

If you have any questions on BMJ Open's open peer review process please email editorial.bmjopen@bmj.com

BMJ Open

Study protocol for a multi-centre, randomized, controlled trial to assess the effectiveness of antimicrobial central venous catheters versus ordinary central venous catheters at reducing catheter-related infections in critically ill Chinese patients

Journal:	*BMJ Open*
Manuscript ID	bmjopen-2017-016564
Article Type:	Protocol
Date Submitted by the Author:	23-Feb-2017

Complete List of Authors:	Wu, Minming; Sichuan University West China Hospital, Medical ICU<br>Chen, Yao; Sichuan University West China Hospital, Medical ICU<br>Du, Bin; Peking Union Medical College Hospital, Medical ICU<br>Kang, Yan; Sichuan University West China Hospital, Medical ICU

**Primary Subject Heading**:	Intensive care
Secondary Subject Heading:	Intensive care, Medical management
Keywords:	catheter related infection, central venous catheterization, multi-center randomized controlled trial

SCHOLARONE™ Manuscripts

For peer review only

Study protocol for a multi-centre, randomized, controlled trial to assess the effectiveness of antimicrobial central venous catheters versus ordinary central venous catheters at reducing catheter-related infections in critically ill Chinese patients

Minming Wu[1], Yao Chen[1], Du Bin[2], Kang Yan[1]

[1] Department of Critical Care Medicine, West China Hospital of Medicine, Sichuan University, No. 37 Guo Xue Xiang, Chengdu, Sichuan, 610041, P.R. China

[2] Medical Intensive Care Unit, Peking Union Medical College Hospital, Peking Union Medical College and Chinese Academy of Medical Sciences, Beijing 100730, China

Corresponding author

Dr Kang Yan

Intensive Care Unit, West China Hospital of Medicine, Sichuan University, Chengdu, Sichuan, 610041, China

Tel.: +86 18980601566

Fax: 85422508

Email: kang_yan_123@163.com

For peer review only

Keywords: central venous catheterization, catheter-related infection, multi-centre randomized controlled trial

Authors' contributions

Dr Minming Wu and Prof Du Bin drafted the manuscript. Dr Kang Yan and Dr Yao Chen critically revised the manuscript. Prof Du Bin and Prof Kang Yan together designed the study. Dr Minming Wu and Dr Yao Chen contributed to the study design and development.

Competing interests

The authors declare that they have no competing financial interests.

Funding

This work is supported by B. Braun Melsungen AG (Melsungen, Germany) through individual research contracts with participating institutions. The funding source has no role in the design or conduct of the trial, data collection, analyses, or manuscript preparation.

Acknowledgments

For peer review only - http://bmjopen.bmj.com/site/about/guidelines.xhtml

This manuscript has been revised by Edanz Editiong. The editorial certificate will be submitted in the supplimental file.

Word count: 2968

For peer review only

For peer review only - http://bmjopen.bmj.com/site/about/guidelines.xhtml

Abstract

Introduction: Catheter use is associated with many complications and is an iatrogenic source of morbidity and mortality in intensive care units. The catheter being studied (Certofix® protect) was developed by B. Braun Melsungen AG (Melsungen, Germany) to reduce the risk for catheter-related infections. This clinical trial will compare the safety and efficiency of Certofix® protect with that of an ordinary Certofix® catheter.

Methods and analysis: This is a prospective, multi-centre, controlled, randomized clinical study comparing two central venous catheters. The main objective is to assess the effectiveness of an antimicrobial central venous catheter for reducing catheter-related bloodstream infections, all-cause mortality, catheter colonization, catheter-related thrombosis, and other catheter-related complications in Chinese adult patients who require dual lumen central venous catheterization for more than 5 days and to determine safety. Twelve to 15 medical centres in China will take part. Participants will receive either the antimicrobial central venous catheter or the ordinary central venous catheter by randomization at recruitment. All outcomes including patient characteristics are to be recorded and analysed. The primary endpoint is the incidence of catheter-related bloodstream infections.

Ethics and dissemination: The Ethics Committee of West China Hospital of Sichuan University has granted ethical approval of this study (27 January 2015). Results will be published in peer-reviewed journals and presented at conferences.

Trial registration: Protocol ID: HC-I-H 1503; ClinicalTrials.gov ID: NCT02645682.

For peer review only - http://bmjopen.bmj.com/site/about/guidelines.xhtml

Strengths and limitations of this study

This is the first multi-centre, randomized study to assess the effectiveness of Certofix® protect in critically ill Chinese adult patients and to determine the relationship between catheter-related bloodstream infections and catheter-related thrombosis. Because of differences in the two catheters being studied, it is not possible to blind the people conducting the research.

For peer review only

For peer review only - http://bmjopen.bmj.com/site/about/guidelines.xhtml

Introduction

Over the past 30 years, central venous catheters (CVC) have been an essential part of the management of critically and chronically ill patients. However, CVC are associated with a variety of complications including mechanical injury, infection, and thrombosis, and can lead to increased hospital costs and longer hospital stays and mortality [1, 2, 3].

Catheter-related bloodstream infections (CRBSI) are one of the most common, lethal, and costly complications in patients with indwelling CVC. In the United States, 78 000 CRBSI occur in hospitals and dialysis units annually, resulting in a mortality rate of 12.3% and a cost of USD 7288–USD 29 156 per case [4].

Studies have reported that CVC coated or impregnated with antimicrobial agents might prevent CRBSI by inhibiting the adherence of microorganisms and the formation of biofilm [5]. A meta-analysis [6] reported a significant reduction in CRBSI (risk ratio [RR]: 0.66, 95% confidence interval [CI]: 0.75–1.05) and catheter colonization (RR: 0.88, 95% CI: 0.75–1.05) in an impregnated catheter group compared with a non-impregnated group, but no difference in systemic infections (RR: 1.0, 95% CI: 0.88–1.13) and all-cause mortality (RR: 0.88, 95% CI: 0.75–1.05).

Catheter-related thrombosis (CRT) is another common complication of long-term indwelling CVC. CRT can cause complications such as pulmonary embolism and infection. Critically ill patients with CRBSI are more likely to get CRT [7, 8].

Although many studies on antimicrobial catheters have been conducted, research in China is limited. Different management approaches to CVC may result in different outcomes

For peer review only - http://bmjopen.bmj.com/site/about/guidelines.xhtml

in catheter-related infection rates, affecting estimates on the effects of antimicrobial catheters. Unfortunately, no study has provided evidence that antithrombotic therapy can prevent CRT.

A new antimicrobial CVC (Certofix® protect) has been developed by B. Braun Melsungen AG (Melsungen, Germany) to reduce the risk for catheter-related infections and CRT. A prospective, randomized, double-bind clinical trial (NCT00555282) conducted in the Czech Republic found that the blood stream infection rate was significantly lower in a protected CVC group compared with a standard CVC group (2.00% *vs.* 6.47%, p=0.008), and that the incidence of blood stream infections/1000 catheter-days was lower for coated catheters (3.21 *vs.* 8.30, p=0.036), but coated CVC had a similar incidence as standard CVC (17.36% *vs.* 18.67%, p=0.747) as well as a similar incidence of CRBSI (1.33% *vs.* 1.94%, p=0.752) [9].

In the current study, all catheters are polyurethane dual-lumen CVC for catheterization of the vena cava according to the Seldinger technique with the possibility of an intra-atrial ECG lead and are manufactured by B. Braun Melsungen AG (Melsungen, Germany). Intervention catheters are antimicrobially modified. Polarization of the catheter material destroys the cell wall structure of microorganisms in case of surface colonization. Perpetual chemical interaction between the polyurethane of the catheter and the agent biguanide ensures the reduction of catheter-related infections during the entire application of the catheter. Control catheters are standard common dual-lumen catheters. The two kinds of catheters are distinguishable in appearance and packaging.

This is a prospective, multi-centre, parallel group, controlled, randomized clinical

For peer review only - http://bmjopen.bmj.com/site/about/guidelines.xhtml

study performed to assess the effectiveness of Certofix® protect at reducing catheter-related infections in critically ill Chinese adult patients who require central venous catheterization. Other objectives are to assess the effectiveness of Certofix® protect in reducing catheter colonization and CRT and the relationship between catheter-related infections and CRT.

Methods

Study design

The recruitment period of the study is 18 months, from April 2016 until December 2017, at 12–16 hospital centres. The intervention group is those patients that undergo catheterization with Certofix® protect. The control group is those patients that undergo catheterization with Certofix®. Patients are prospectively followed from the day of CVC insertion for at least 5 days or up until CVC removal, whichever comes first. Inclusion criteria are: (1) adult patients (>18 years) admitted to an intensive care unit; (2) dual-lumen CVC; (3) patients expected to require indwelling catheterization for at least 5 days; and (4) patients who provide signed informed consent. Peripherally inserted venous catheters, peripherally inserted arterial catheters (including FloTrac®), femoral arterial catheters (including PiCCO®), haemodialysis, pulmonary arterial catheters, and peripherally inserted central catheters can be used in the study. Except for the catheters mentioned above, any other catheter is not permitted. Exclusion criteria are: (1) pregnant women or women who have recently given birth; (2) patients with malignant diseases and unlikely to survive for the next 28 days in the opinion of the intensive care unit consultant; (3) patients with suspected catheter-related

For peer review only - http://bmjopen.bmj.com/site/about/guidelines.xhtml

infections; (4) patients receiving an initial study catheter through guidewire exchange; (5) patients hospitalised for severe burn injuries; (6) patients with, in the opinion of the doctors, a situation that is not suitable for indwelling, including allergy to the material of the catheter, confirmed deep vein thrombosis, chronic inflammatory skin disorders at the catheter insertion site, coagulation dysfunction (such as antithrombotic prophylaxis), abnormal anatomical structure (enlargement of thyroid glands, cervical tumours, severe pneumonectasis, post-surgical changes of the insertion site); (7) patients who have been enrolled in the study before (during hospitalization); and (8) patients enrolled in another investigative trial in the past 3 months.

Table 1 shows a schedule for participant enrolment, interventions, assessments, and visits. During treatment, doctors are required to collect data and samples from patients and arrange tests. All notices are provided in Supplemental File 1.

Study endpoints

The primary endpoint is CRBSI. CRBSI is defined as an isolate of an organism from a quantitative or semi-quantitative culture of a distal catheter segment and from a separate percutaneous blood culture, or an isolate of an organism from a blood culture from the catheter and from a separate percutaneous blood culture, with a time interval of two positive outcomes in more than 2 hours. Clinicians should make sure the infection cannot be from another identifiable source of infection. The secondary endpoints are catheter colonization; attack rate of CRT (insertion side or contralateral side); morbidity of CRT (insertion side or

For peer review only - http://bmjopen.bmj.com/site/about/guidelines.xhtml

contralateral side); and hospital mortality. Catheter colonization is defined as any positive semi-quantitative culture of a distal catheter segment using the roll-plate method (Marki method).

Study population

The study sample size is calculated on the basis of an expected CRBSI rate of approximately 3% for the control group and 6% for the antiseptic catheter group. Allowing for a 10% dropout rate, 1818 patients are required to yield a study with 80% power at a statistical significance level of 0.05.

Participant selection and recruitment

Before identifying and screening patients for eligibility, informed consent must be obtained by the doctor in charge (Figure 1). All information is to be transferred into an electronic database so that the trial office can monitor recruitment and refusal rates at each centre.

Randomization

For a patient who meets the required criteria, a researcher opens a randomized card that records the screening number and treatment allocation group for random allocation of the patients. To ensure that patients are randomly assigned at a 1:1 ratio at each study centre, the

For peer review only - http://bmjopen.bmj.com/site/about/guidelines.xhtml

For peer review only

randomized cards should be protected using a block design.

Treatment allocation (blinding)

As the two kinds of CVC under investigation are different in some details, it is impossible to blind local investigative doctors. Outcome data assessment and analysis will be posted to participants by the coordinating centre, which will be blind.

Patient termination and withdrawal criteria

Participants and their authorised surrogates will participate in the study voluntarily, therefore, they may withdraw from the trial at any time for any reason. Patients may also be withdrawn from the study for: (1) severe adverse events; or (2) violating or deviating from the protocol. If a patient is withdrawn for one of the two reasons mentioned, they should proceed to security analysis.

Research centre termination and withdrawal criteria

A research centre must terminate their involvement in the clinical trial if: (1) the researchers do not obey the rules of the International Conference on Harmonisation Guidelines for Good Clinical Practice or local regulations; (2) the research centre intentionally submits incorrect or incomplete data to inspectors; (3) the requirements of the protocol are not met, including poor

For peer review only - http://bmjopen.bmj.com/site/about/guidelines.xhtml

For peer review only

data quality (incomplete case report forms); or (4) investigators make changes without informing the lead researchers. Each investigator should be qualified and be approved by the lead researchers. As a 10% dropout rate is allowed, there will be no need to add new patients when an existing participant withdraws from the trial.

Data collection and inspection

Data collection begins on the day a participant signs the informed consent and continues until the participant is discharged or transferred to another hospital. Data are collected using a paper-based case report form and an electronic database.

Investigators follow a schedule to collect data, including: (1) screening data, informed consent, demographic data, inclusion and exclusion criteria, and enrolment data; (2) baseline information on catheterization, vascular ultrasound of veins at the insertion site and contralateral site, and CVC catheterization; (3) CVC removal data, peripheral blood cultures, catheter blood cultures, catheter tip cultures, and vascular ultrasound of veins at the insertion site and contralateral site; and (4) prognosis, date of transferring out of the intensive care unit, and date of discharge/death, whichever comes first.

Follow-up data

Statistical analysis plan

A detailed statistical analysis plan setting out full details of the proposed analyses will be

For peer review only - http://bmjopen.bmj.com/site/about/guidelines.xhtml

completed before the trial database is locked for analyses. If researchers make adjustments to the protocol before the statistical analysis plan is determined, the amendment will be added to the plan.

Hypothesis

The study hypothesis is:

H_0: πCVCp = πCVC

H_1: πCVCp < πCVC

Where π represents the incidence of CRBSI.

Analysis sets

There will be a full analysis set, a per protocol set, and a safety set.

Proposed interim analysis

An interim analysis will be conducted in the middle of the recruitment period to evaluate the effectiveness of the main indexes and to determine whether it is necessary/possible to terminate the trial early. An independent data safety monitoring board has been established to oversee the safety of the trial participants and may suggest terminating the study when the

For peer review only - http://bmjopen.bmj.com/site/about/guidelines.xhtml

outcome of the interim analysis reaches the determined threshold. Table 2 shows the alpha spending functions and cutoff values.

Statistical analysis

Principles

All statistical tests will be two tailed and will be analysed using SAS statistical analysis software (ver. 9.4; SAS Institute, Cary, NC). Quantitative variables will be analysed by calculating the mean, standard deviation, median, minimum value, maximum value, lower quartile (Q1), and upper quartile (Q3). Categorical variables will be described using cases and percentages for each category. The significance of differences between two groups will be determined using the chi-square test or Fisher's exact test for categorical data, the group *t*-test or Wilcoxon rank sum test for continuous data, and the Wilcoxon rank sum test or the Cochran–Mantel–Haenszel chi-square test for ranked data.

Proposed primary analysis

The incidence rates of CRBSI in the two groups will be compared using the Cochran–Mantel–Haenszel chi-square test. For the interim analysis, the size of the test for α_1 is 0.003, and we will also calculate $(1-\alpha_1) \times 100\%$ confidence intervals. If the result rejects H_0, then the antimicrobial CVC group is superior to the ordinary CVC group. If the interim analysis shows no statistical significance or if the data safety monitoring board decides to

For peer review only - http://bmjopen.bmj.com/site/about/guidelines.xhtml

complete the next stage of the trial, then we will complete the final analysis (α_2 = 0.049, CI ($1-\alpha_2$) × 100%). If the result rejects H_0, then the antimicrobial CVC group is superior to the ordinary CVC group. The proposed primary analysis is based on the final analysis set and the per protocol set.

Other planned analyses

Catheter colonization, rate of CRT, and hospital mortality in the two groups will be compared using the chi-square test or Fisher's exact test. Analyses of the other indicators follows the process described under "Principles" above. Analyses of the secondary indicators is based on the full analysis set and the per protocol set.

Missing data

Worst-case imputation will be used to evaluate missing data in the full analysis set. Dropout rates will be obtained and for each group we will determine if the dropout rate is higher than the difference in event rates between the two groups using the worst-case scenario model.

Author independence

The study authors affiliated with the West China Hospital and the Peking Union Medical College Hospital designed the study. The authors have full independence in decisions

For peer review only - http://bmjopen.bmj.com/site/about/guidelines.xhtml

regarding the reporting of results and the content of the reported study.

Safety evaluation

The proportion of abnormal cases after treatment will be determined, as will the number of cases/incidence of adverse events and severe adverse events. We will also describe the clinical manifestations, degrees of all adverse events, and the relationship between these factors and the catheters in detail. Changes in indexes will be described using a crosstab grid. All safety evaluations will be based on the safety set.

Adverse events

Definitions

An adverse event is defined as a patient who develops clinical features such as discomfort or laboratory abnormalities that are not related to the expected therapeutic effects during central venous catheterization.

The catheter-associated adverse events to be recorded are: (1) a broken or cracked catheter; (2) hematoma at the insertion site; (3) chylothorax, pneumothorax, haemothorax, or pleural effusion caused by mispuncture or malposition; (4) arrhythmia or rupture of the atrium caused by malposition, endocarditis because of mechanical stimulation, thrombophlebitis, or injury to the atrium, thoracic duct, brachial plexus, or phrenic nerve because of mispucture.

For peer review only - http://bmjopen.bmj.com/site/about/guidelines.xhtml

The severe adverse events (definitely related or possibly related) to be recorded are: (1) death as the result of an adverse event. Medical conditions resulting in death need to be comprehensively reported, such as an underlying disease or an accident; (2) life-threatening events. Life-threatening events are those events that put the patient at risk for death at the time. This is distinct from an event that may become more serious in the future and put the patient at risk for death; (3) events requiring hospitalization or prolong the time of hospitalization. Hospitalization in this context means more than 1 calendar day; (4) events leading to permanent damage, or medical intervention that must be taken to avoid permanent damage.

An event may meet more than one criteria. If the event could results in harm to a patient or clinician, intervention should be taken to prevent the event, and this adverse event should be recorded as a severe adverse event.

Recording and reporting

Researchers must record adverse events and severe adverse events in the corresponding case report form, including signs and symptoms, date, disappearance date (duration), severity or strength, relationship with therapy, measurements, and outcomes. If the interim analysis finds that the morbidity of some type of adverse event or severe adverse event and its severity increases significantly, researchers must report the adverse event in a timely manner. All severe adverse events must be reported to the drug administration department and the ethics committee within 24 hours (one working day), and the production enterprise must be

For peer review only - http://bmjopen.bmj.com/site/about/guidelines.xhtml

informed at the same time.

Follow-up

Researchers must follow-up all adverse events and severe adverse events during the trial. Follow-up will continue until the adverse event or the severe adverse event disappears or becomes stable. All adverse events are to be kept in the case report form until the last observation date.

Quality control

Quality control is defined as “a part of quality management focused on fulfilling quality requirements” [10]. This approach places an emphasis on three aspects: (1) Elements: such as controls, job management, defined and well-managed processes [11], performance and integrity criteria, and identification of records; (2) Competence: such as knowledge, skills, experience, and qualifications; (3) Soft resources: such as personnel, integrity, confidence, organizational culture, motivation, team spirit, and quality relationships. In study management, quality control requires that the project manager and the team inspect the work to ensure its alignment with the project scope [12].

Study inspection

For peer review only - http://bmjopen.bmj.com/site/about/guidelines.xhtml

Authorised and qualified researchers will visit the research centres to verify adherence to the protocol and regulations, ensure original data, and to assist research activities according to the inspection plan.

Ethics and dissemination

The protocol has been registered at the ClinicalTrials.gov registry. Any revisions to the protocol will be documented in the ClinicalTrials.gov registry. Written informed consent will be obtained from all participants. We will publish the results of this trial in peer-reviewed clinical journals and present the findings at conferences for widespread dissemination of the results.

Trial status

Data collection is ongoing.

For peer review only - http://bmjopen.bmj.com/site/about/guidelines.xhtml

For peer review only

References

1. O'Grady N, Alexander M, Burns LA, et al. Guidelines for the prevention of intravascular catheter-related infections, 2011. Am J Infect Control 2011; 39(4 Suppl 1): S1-34

2. Cicalini S, Palmieri F, Petrosillo N. Clinical review: New technologies for prevention of intravascular catheter-related infections. Crit Care 2004; 8: 157-62

3. Saint S, Veenstra DL, Lipsky BA. The clinical and economic consequences of nosocomial central venous catheter-related infection: are antimicrobial catheters useful? Infection Control and Hospital Epidemiology 2000; 21: 375-80

4. Rupp ME. Central venous catheters coated or impregnated with antimicrobial agents effectively prevent microbial colonisation and catheter-related bloodstream infections. Evid Based Med. 2014; 19: 56

5. Halton KA, Cook DA, Whitby M, et al. Cost effectiveness of antimicrobial catheters in the intensive care unit: addressing uncertainty in the decision. Crit Care 2009; 13: R35

6. Lai NM, Chaiyakunapruk N, Lai NA. Catheter impregnation, coating or bonding for reducing central venous catheter-related infections in adults. Cochrane Database Syst Rev 2013; 6: CD007878

7. Mehall JR, Salzman DA, Jackson JR, et al. Fibrin sheath enhances central venous catheter infection. Crit Care Med 2002; 30: 908-12

8. Raad I, Khalil S, Coserton JW, et al. The relationship between the thrombotic and

For peer review only

infectious complications of central venous catheters. JAMA 1994; 271: 1014-6

9. http://braunoviny.bbraun.cz/clanky/polyhexanide-anti-infective-coating-ofcentral-venous/ 10. ISO 9000:2005, Clause 3.2.10

11. Dennis Adsit (November 23, 2007). “What the Call Center Industry Can Learn from Manufacturing: Part II” (PDF). National Association of Call Centers. Retrieved 21 December 2012.

12. Phillips, Joseph (November 2008). “Quality Control in Project Management”. The Project Management Hut. Retrieved 21 December 2012.

13. Maki DG, Stoltz SM, Wheeler S, et al. Prevention of central venous catheter-related bloodstream infection by use of an antiseptic-impregnated catheter: A randomized, controlled trial. Ann Intern Med1997; 127: 257-66

14. Raad I, Darouiche R, Dupuis J, et al. Central venous catheters coated with minocycline and rifampin for the prevention of catheter-related colonization and bloodstream infections: A randomized, double-blind trial. Ann Intern Med 1997; 127: 267-74

15. Rupp ME, Lisco SJ, Lipsett PA, et al. Effect of a second generation venous catheter impregnated with chlorhexidine and sliver sulfadiazine on central catheter-related infections: A randomized, controlled trial. Ann Intern Med 2005; 143: 570-80

16. Gong P, Li H, He X, et al. Preparation and antibacterial activity of Fe3O4@Ag nanoparticles. Nanotechnology 2007; 18: 604-11

For peer review only - http://bmjopen.bmj.com/site/about/guidelines.xhtml

17. Shrivastava S, Bera T, Roy A, et al. Characterization of enhanced antibacterial effects of novel silver nanoparticles. Nanotechnology 2007; 18: 225103-225112

18. Samuel U, Guggenbichler JP. Prevention of catheter-related infections: the potential of a new nano-silver impregnated catheter. Int J Antimicrob Agents 2004; 23: 75-8

19. Hsu SH, Tseng HJ, Lin YC. The biocompatibility and antibacterial properties of waterborne polyurethane-silver nanocomposites. Biomaterials 2010; 31: 6796-808

20. Campisi C, Biffi R, Pittiruti M. Catheter-related venous thrombosis: the development of a nationwide consensus paper in Italy. J AssocVasc Access 2007; 12: 38-46

21. Cortelezzi A, Moia M, Falanga A. Incidence of thrombotic complications in patients with haematological malignancies with central venous catheters: a prospective multicenter study. Br J Haematol 2005; 129: 811-7

22. Monreal M, Raventos A, Lerma R, et al. Pulmonary embolism in patients with upper extremity DVT associated to venous central lines–a prospective study. ThrombHaemost1994; 72: 548-50

23. Kearon C, Akl EA, Comerota AJ, et al. Antithrombotic therapy for VTE disease: Antithrombotic Therapy and Prevention of Thrombosis. American College of Chest Physicians Evidence-Based Clinical Practice Guidelines. 9th Ed. Chest2012; 141(Suppl 2): e419S–94S

24. Pierce CM, Wade A, Mok Q, et al. Heparin-bonded central venous lines reduce thrombotic and infective complications in critically ill children. Intensive Care Med

For peer review only - http://bmjopen.bmj.com/site/about/guidelines.xhtml

2000; 26: 967-72

25. http://www.safeinfusiontherapy.com/documents/french/Certofix_Brochure.pdf

26. U.S. Department of Health and Human Services. Guidance for Industry E9, Statistical Principles for Clinical Trials. 1998.

For peer review only

For peer review only - http://bmjopen.bmj.com/site/about/guidelines.xhtml

Table 1 Time to visit and data collection

	Enrollment	Allocation	Post allocation	Closeout
Informed consent	×			
Inclusion/exclusion criteria	×			
Randomization	×			
Medical history & physical examination	×			
Temperature		×	×	
Insertion		×		
Blood test		×	×	
Blood culture			×	
Culture of CVC			×	
Vein ultrasound		×	×	
AE/SAE	×	×	×	×
Treatment/drug combination	×	×	×	×

Table 2 Alpha spending function and cut off value

	Lower bound	Upper bound	Alpha size of test	Alpha spending	Cumulative alpha	Power of test	Overall efficiency
Interim analysis	-2.96259	2.96259	0.003051	0.003051	0.003051	0.164276	0.164276
Final analysis	-1.96857	1.96857	0.049002	0.046949	0.050000	0.636018	0.800294

The distribution of suspension boundary (alpha) is normal distribution.

For peer review only

Training before trail

1) Procedures for insertion

First doctors chose a proper insertion site, and then used maximal barrier precautions during insertion (the operator was required to wear masks, sterile gloves, and surgical gowns and use large sterile drapes). After disinfected with povidone iodine or chlorhexidine, the catheter was inserted percutaneously using Seldinger technique. It was not allowed to exchange the catheter over a guidewire into an old site. Sites were dressed with hyalo-dressing.

2) Care of the catheter during indwelling catheterization

Twice a week or according to routine procedures, perform the follows: the dressing removed; the site inspected and cleaned with povidone-iodine or chlorhexidine; and the new dressing applied.

3) Remove catheters

At removal, the site was again disinfected by povidone iodine or chlorhexidine to make sure that the skin around the catheter was clean.

4) Indication of removal

No need for CVC in patients; Occlusion of catheter; Suspected or confirmed deep vein thrombosis of insertion site; Patients with highly suspected CRBSI and meeting one of the following criteria, haemodynamic instability, bacteremia; or the doctor in charge insisting to remove the catheter after 5 days' observation.

5)Tests

Blood culture

For the dual-lumen catheter, blood samples were taken from both lumens separately. Researchers should insert percutaneously to take sample from peripheral blood vessels. Aerobic culture and anaerobic culture were needed for each blood sample.

Cultures of CVC

The entire catheter was removed aseptically, and 4-cm segment was cut from the catheter tip, which was semi-quantitatively cultured using the roll-plate method.

Vein ultrasound

It is used to diagnose or to exclude deep vein thrombosis (DVT). If the insertion site is femoral vein, doctors will screen iliac vein and femoral vein on both sides for DVT. While in the jugular vein, bilateral jugular veins should be inspected. Ultrasound is needless only in case of catheterization in subclavian vein. Ultrasound will be arranged before insertion and after withdrawal of catheter (within 48h).

For peer review only

Checklist

	Section/item	Page number
✔	Title	1
✔	Trial registration	1
✔	Protocol version	Supplemental file 3
✔	Funding	1
✔	Roles and responsibilities	1
	Introduction	
✔	Background and rational	2
✔	Objectives	2
✔	Trial design	2
	Methods	
	Participants, interventions, and outcomes	
✔	Study setting	3
✔	Eligibility criteria	3
✔	Interventions	3
✔	Outcomes	3
✔	Participant timeline	Supplemental file 1-Time to visit and data collection
✔	Sample size	4
✔	Recruitment	4
✔	Assignment of interventions (for controlled trials)	4
✔	Allocation sequence	4

For peer review only

	Data collection, management, and analysis	
✔	Data collection methods	4
✔	Data management	4
✔	Statistical methods	5
	Monitoring	
✔	Data monitoring	7
✔	Harm	6
✔	Auditing	4,7
✔	Ethic and dissemination	7
✔	Research ethics approval	7
✔	Protocol amendments	7
✔	Consent or assent	4
✔	Confidentiality	4
✔	Declaration of interests	1,6
✔	Access to data	1
✔	Ancillary and post-trial care	4,6
✔	Dissemination policy	4
	Appendices	
✔	Informed consent materials	Please see supplemental file 4
✔	Biological specimens	Please see supplemental file 2-Training before trail

For peer review only

For peer review only

Study protocol for a multi-centre, randomized, controlled trial to assess the effectiveness of antimicrobial central venous catheters versus ordinary central venous catheters at reducing catheter-related infections in critically ill Chinese patients

Journal:	*BMJ Open*
Manuscript ID	bmjopen-2017-016564.R1
Article Type:	Protocol
Date Submitted by the Author:	14-Aug-2017
Complete List of Authors:	Wu, Minming; Sichuan University West China Hospital, Medical ICU<br>Chen, Yao; Sichuan University West China Hospital, Medical ICU<br>Du, Bin; Peking Union Medical College Hospital, Medical ICU<br>Kang, Yan; Sichuan University West China Hospital, Medical ICU
**Primary Subject Heading**:	Intensive care
Secondary Subject Heading:	Intensive care, Medical management
Keywords:	catheter related infection, central venous catheterization, multi-center randomized controlled trial

SCHOLARONE™ Manuscripts

For peer review only

Study protocol for a multi-centre, randomized, controlled trial to assess the effectiveness of antimicrobial central venous catheters versus ordinary central venous catheters at reducing catheter-related infections in critically ill Chinese patients

Minming Wu[1], Yao Chen[1], Du Bin[2], Kang Yan[1]

[1] Department of Critical Care Medicine, West China Hospital of Medicine, Sichuan University, No. 37 Guo Xue Xiang, Chengdu, Sichuan, 610041, P.R. China

[2] Medical Intensive Care Unit, Peking Union Medical College Hospital, Peking Union Medical College and Chinese Academy of Medical Sciences, Beijing 100730, China

Corresponding author

Dr Kang Yan

Intensive Care Unit, West China Hospital of Medicine, Sichuan University, Chengdu, Sichuan, 610041, China

Tel.: +86 18980601566

Fax: 85422508

Email: kang_yan_123@163.com

For peer review only - http://bmjopen.bmj.com/site/about/guidelines.xhtml

Keywords: central venous catheterization, catheter-related infection, multi-centre randomized controlled trial

Authors' contributions

Prof Du Bin and Prof Kang Yan together designed the study. Dr Minming Wu and Prof Du Bin drafted the manuscript. Dr Kang Yan and Dr Yao Chen critically revised the manuscript. Dr Minming Wu and Dr Yao Chen contributed to the study design and development.

Competing interests

The authors declare that they have no competing financial interests.

Funding

This work is supported by B. Braun Melsungen AG (Melsungen, Germany) through individual research contracts with participating institutions. The funding source has no role in the design or conduct of the trial, data collection, analyses, or manuscript preparation.

Acknowledgments

For peer review only

This manuscript has been revised by Edanz Editing.

Word count: 2697

For peer review only

For peer review only - http://bmjopen.bmj.com/site/about/guidelines.xhtml

Abstract

Introduction: Catheter use is associated with many complications and is an iatrogenic source of morbidity and mortality in intensive care units (ICU). The catheter being studied (Certofix® protect) was developed to reduce the risk for catheter-related infections. This clinical trial will compare the safety and efficiency of Certofix® protect with that of an ordinary Certofix® catheter.

Methods and analysis: In this multicenter trial, we randomly assigned dual lumen central venous catheterization (≥5ds) in patients in the adult ICU to the antimicrobial CVC group or ordinary CVC group. We planed to recruit 12 to 16 medical centers in China. Our main objective was to assess the effectiveness of antimicrobial CVCs in reducing CRBSI, all-cause mortality, catheter colonization, CRT and other catheter related complications. The primary outcome was the incidence of catheter-related bloodstream infection (CRBSI).

Ethics and dissemination: The Ethics Committee of West China Hospital of Sichuan University has granted ethical approval of this study (27 January 2015). Results will be published in peer-reviewed journals and presented at conferences.

Trial registration: Protocol ID: HC-I-H 1503; ClinicalTrials.gov ID: NCT02645682.

Strengths and limitations of this study

This is the first multi-centre, randomized study to assess the effectiveness of Certofix® protect in critically ill Chinese adult patients and to determine the relationship between

For peer review only - http://bmjopen.bmj.com/site/about/guidelines.xhtml

catheter-related bloodstream infections and catheter-related thrombosis. Because of differences in the two catheters being studied, it is not possible to blind the people conducting the research. Whether the local physician use the ultrasound as guidance may influence major outcomes even though we recommend experienced operators to conduct the insertion. It's difficult to distinguish and exclude patients whose expected survival less than one month cause their condition are constantly changing. What's more, ICU patients suffer from many underlying diseases which makes difficult to judge from the source of infection.

For peer review only

For peer review only - http://bmjopen.bmj.com/site/about/guidelines.xhtml

Introduction

Over the past 30 years, central venous catheters (CVC) have been an essential part of the management of critically and chronically ill patients. However, CVC are associated with a variety of complications including mechanical injury, infection, and thrombosis, and can lead to increased hospital costs and longer hospital stays and mortality [1, 2, 3].

Catheter-related bloodstream infection (CRBSI) is one of the most common, lethal, and costly complications in patients with indwelling CVC [4]. Studies have reported that CVC coated or impregnated with antimicrobial agents could reduce CRBSI and catheter colonization, but didn't reduce systemic infections and all-cause mortality [5 - 13]. Catheter-related thrombosis (CRT) is another common complication of long-term indwelling CVC [14 - 18]. CRT can cause complications such as pulmonary embolism and infection. Critically ill patients with CRBSI are more likely to get CRT [19, 20]. Although many studies on antimicrobial catheters, CRT and relationship between them have been conducted, research in China is limited.

We conducted this multi-centre study to assess the effectiveness of Certofix® protect (supplemental appendix – study catheter) at reducing CRBSI, catheter colonization and CRT in critically ill Chinese adult patients. We also try to find out the relationship between catheter-related infections and CRT.

Methods

For peer review only - http://bmjopen.bmj.com/site/about/guidelines.xhtml

Study design

This is a prospective, multi-centre, parallel group, controlled, randomized clinical trial conducted at 12-16 hospital centres of China from April 2016 until December 2017. The Ethics Committee of West China Hospital of Sichuan University has granted ethical approval of this study (27 January 2015).

Eligibility criteria

Inclusion criteria are: (1) adult patients (>18 years) admitted to an intensive care unit; (2) dual-lumen CVC; (3) patients expected to require indwelling catheterization for at least 5 days; and (4) patients who provide signed informed consent. Peripherally inserted venous catheters, peripherally inserted arterial catheters (including FloTrac®), femoral arterial catheters (including PiCCO®), haemodialysis, pulmonary arterial catheters, and peripherally inserted central catheters can be used in the study. Except for the catheters mentioned above, any other catheter is not permitted.

Exclusion criteria are: (1) pregnant women or women who have recently given birth; (2) patients with malignant diseases and unlikely to survive for the next 28 days in the opinion of the intensive care unit consultant; (3) patients with suspected catheter-related infections; (4) patients receiving an initial study catheter through guidewire exchange; (5) patients hospitalised for severe burn injuries; (6) patients with, in the opinion of the doctors, a situation that is not suitable for indwelling, including allergy to the material of the catheter, confirmed deep vein thrombosis, chronic inflammatory skin disorders at the catheter insertion site, coagulation dysfunction (such as antithrombotic prophylaxis), abnormal anatomical

For peer review only - http://bmjopen.bmj.com/site/about/guidelines.xhtml

structure (enlargement of thyroid glands, cervical tumours, severe pneumonectasis, post-surgical changes of the insertion site); (7) patients who have been enrolled in the study before (during hospitalization); and (8) patients enrolled in another investigative trial in the past 3 months.

The intervention group is those patients that undergo catheterization with Certofix® protect. The control group is those patients that undergo catheterization with Certofix®. Patients are prospectively followed from the day of CVC insertion for at least 5 days or up until CVC removal, whichever comes first. Table 1 shows a schedule for participant enrolment, interventions, assessments, and visits. During treatment, local investigators are required to collect data and samples from patients and arrange tests. All notices are provided in Supplemental appendix.

Table 1 Time to visit and data collection

	Enrollment	Allocation	Post allocation	Closeout
Informed consent	×			
Inclusion/exclusion criteria	×			
Randomization	×			
Medical history & physical examination	×			
Temperature		×	×	
Insertion		×		
Blood test		×	×	
Blood culture			×	
Culture of CVC			×	
Vein ultrasound		×	×	
AE/SAE	×	×	×	×
Treatment/drug combination	×	×	×	×

For peer review only - http://bmjopen.bmj.com/site/about/guidelines.xhtml

Study endpoints

The primary endpoint is CRBSI. CRBSI [21] is defined as CVC-tip colonization by quantitative or semi-quantitative method and at least one peripheral blood culture positive for the same microorganism or differential time to positivity (>120 min from central and peripheral blood culture). Clinicians should make sure the infection cannot be from another identifiable source of infection. Each suspect case should be discussed with chief doctor of medical group. The secondary endpoints are catheter colonization; attack rate of CRT (insertion side or contralateral side); morbidity of CRT (insertion side or contralateral side); and hospital mortality. Catheter colonization [21] is defined as any positive semi-quantitative culture of a distal catheter segment using the roll-plate method (Maki method). The detail description of how and when outcome measures are defined in supplemental appendix – supplemental method.

Study population

The study sample size is calculated on the basis of an expected CRBSI rate of approximately 6% for the control group and 3% for the antiseptic catheter group. Allowing for a 10% dropout rate, 1818 patients are required to yield a study with 80% power at a statistical significance level of 0.05.

For peer review only - http://bmjopen.bmj.com/site/about/guidelines.xhtml

Participant selection and recruitment

Before identifying and screening patients for eligibility, informed consent (supplemental file) must be obtained by the doctor in charge. All information is to be transferred into an electronic database so that the trial office can monitor recruitment and refusal rates at each centre.

Randomization

Each research centre will receive sequentially numbered containers used to implement the random allocation sequence. And the treatment allocation group is hiding beyond the coated card. To ensure that patients are randomly assigned at a 1:1 ratio at each study centre, the randomized cards was protected using a block design (each block includes 4 random allocation sequence). For a patient who meets the required criteria, the local investigator opens a randomized card that records the screening number and treatment allocation group. Then, physician in charge of the patient will obtain the right study catheter and complete catheterization. So that treatment allocation is concealed.

Patient termination and withdrawal criteria

Participants and their authorised surrogates will participate in the study voluntarily, therefore, they may withdraw from the trial at any time for any reason. Patients may also be withdrawn from the study for: (1) severe adverse events; or (2) violating or deviating from the protocol.

For peer review only - http://bmjopen.bmj.com/site/about/guidelines.xhtml

If a patient is withdrawn for one of the two reasons mentioned, they should proceed to security analysis.

Research centre termination and withdrawal criteria

A research centre must terminate their involvement in the clinical trial if: (1) the researchers do not obey the rules of the International Conference on Harmonisation Guidelines for Good Clinical Practice or local regulations; (2) the research centre intentionally submits incorrect or incomplete data to inspectors; (3) the requirements of the protocol are not met, including poor data quality (incomplete case report forms); or (4) investigators make changes without informing the lead researchers. Each investigator should be qualified and be approved by the lead researchers. As a 10% dropout rate is allowed, there will be no need to add new patients when an existing participant withdraws from the trial.

Data collection and inspection

Principal investigators will centralize all the data monthly and send a newsletter to each centre to promote data quality and process of the trial. Data collection begins on the day a participant signs the informed consent and continues until the participant is discharged or transferred to another hospital. Data are collected using a paper-based case report form (supplemental file, data collection form) and an electronic database.

Investigators follow a schedule to collect data, including: (1) screening data, informed

For peer review only - http://bmjopen.bmj.com/site/about/guidelines.xhtml

consent, demographic data, inclusion and exclusion criteria, and enrolment data; (2) baseline information on catheterization (age, male, ID, height, weight, risk factor of infection, SOFA score, APACH2 score, underlying diseases, antibiotic therapy), vascular ultrasound of veins at the insertion site and contralateral site, and CVC catheterization (date, temperature, catheter type, insertion site, Neutrophil count, antibiotic therapy, other type of catheterization, SAE); (3) CVC removal data (duration of catheterization, temperature, reason for catheter removal, parenteral nutrition, Neutrophil count), peripheral blood cultures, catheter blood cultures, catheter tip cultures, and vascular ultrasound of veins at the insertion site and contralateral site; and (4) prognosis, date of transferring out of the intensive care unit, and date of discharge/death, whichever comes first.

Follow-up data

Statistical analysis plan

Hypothesis

The study hypothesis is:

H_0: πCVCp = πCVC

H_1: πCVCp < πCVC

Where π represents the incidence of CRBSI.

For peer review only - http://bmjopen.bmj.com/site/about/guidelines.xhtml

Analysis sets

There will be a full analysis set, a per protocol set, and a safety set (supplemental appendix – supplemental method).

Statistical analysis

Principles

All statistical tests will be two tailed and will be analysed using SAS statistical analysis software (ver. 9.4; SAS Institute, Cary, NC). Quantitative variables will be analysed by calculating the mean, standard deviation, median, minimum value, maximum value, lower quartile (Q1), and upper quartile (Q3). Categorical variables will be described using cases and percentages for each category. The significance of differences between two groups will be determined using the chi-square test or Fisher's exact test for categorical data, the group *t*-test or Wilcoxon rank sum test for continuous data, and the Wilcoxon rank sum test or the Cochran–Mantel–Haenszel chi-square test for ranked data.

Proposed primary analysis

The incidence density of CRBSI in the two groups will be compared using the Cochran–Mantel–Haenszel chi-square test or Cox model. For the interim analysis, the size of the test for α_1 is 0.003, and we will also calculate $(1-\alpha_1) \times 100\%$ confidence intervals. If the result rejects H_0, then the antimicrobial CVC group is superior to the ordinary CVC group. If

For peer review only - http://bmjopen.bmj.com/site/about/guidelines.xhtml

the interim analysis shows no statistical significance or if the data safety monitoring board decides to complete the next stage of the trial, then we will complete the final analysis (α_2 = 0.049, CI (1-α_2) × 100%). The proposed primary analysis is based on the final analysis set and the per protocol set. Table 2 shows the alpha spending functions and cut-off values.

Table 2 Alpha spending function and cut off value

	Lower bound	Upper bound	Alpha size of test	Alpha spending	Cumulative alpha	Power of test	Overall efficiency
Interim analysis	-2.96259	2.96259	0.003051	0.003051	0.003051	0.164276	0.164276
Final analysis	-1.96857	1.96857	0.049002	0.046949	0.050000	0.636018	0.800294

The distribution of suspension boundary (alpha) is normal distribution.

Secondary analysis

Incidence density of Catheter-tip colonization, CRT, and hospital mortality in the two groups will be compared using the chi-square test or Fisher's exact test, or random-intercept logistic regression. Analyses of the other indicators follows the process described under "Principles" above. Analyses of the secondary indicators is based on the full analysis set and the per protocol set.

Subgroup analysis

Subgroup analyses will be conducted for predefined factors such as insertion site, catheter durations, antibiotic therapy, anticoagulation therapy, underlying diseases, BMI, SOFA score,

For peer review only - http://bmjopen.bmj.com/site/about/guidelines.xhtml

APACHE2 score, etc. Other exploratory subgroup analyses will be eventually conducted.

Safety analysis

The proportion of abnormal cases after treatment will be determined, as will the number of cases/incidence of adverse events and severe adverse events. We will also describe the clinical manifestations, degrees of all adverse events, and the relationship between these factors and the catheters in detail. Changes in indexes will be described using a crosstab grid. All safety evaluations will be based on the safety set.

Missing data

Worsts Observation Carried Forward will be used to evaluate missing data in the full analysis set. Dropout rates will be obtained and for each group we will determine if the dropout rate is higher than the difference in event rates between the two groups using the worst-case scenario model.

Proposed interim analysis

An interim analysis will be conducted in the middle of the recruitment period to evaluate the effectiveness of the main indexes and to determine whether it is necessary/possible to

For peer review only - http://bmjopen.bmj.com/site/about/guidelines.xhtml

terminate the trial early.

Adverse events

Definitions

An adverse event is defined as a patient who develops clinical features such as discomfort or laboratory abnormalities that are not related to the expected therapeutic effects during central venous catheterization.

The catheter-associated adverse events according to the modified CTCAE V.4 classification to be recorded are: (1) a broken or cracked catheter; (2) hematoma at the insertion site; (3) chylothorax, pneumothorax, haemothorax, or pleural effusion caused by mispuncture or malposition; (4) arrhythmia or rupture of the atrium caused by malposition, endocarditis because of mechanical stimulation, thrombophlebitis, or injury to the atrium, thoracic duct, brachial plexus, or phrenic nerve because of mispuncture.

The severe adverse events (definitely related or possibly related) to be recorded are: (1) death as the result of an adverse event. Medical conditions resulting in death need to be comprehensively reported, such as an underlying disease or an accident; (2) life-threatening events. Life-threatening events are those events that put the patient at risk for death at the time. This is distinct from an event that may become more serious in the future and put the patient at risk for death; (3) events requiring hospitalization or prolong the time of

For peer review only - http://bmjopen.bmj.com/site/about/guidelines.xhtml

hospitalization. Hospitalization in this context means more than 1 calendar day; (4) events leading to permanent damage, or medical intervention that must be taken to avoid permanent damage.

An event may meet more than one criteria. If the event could result in harm to a patient or clinician, intervention should be taken to prevent the event, and this adverse event should be recorded as a severe adverse event.

Recording and reporting

Researchers must record adverse events and severe adverse events in the corresponding case report form, including signs and symptoms, date, disappearance date (duration), severity or strength, relationship with therapy, measurements, and outcomes. If the interim analysis finds that the morbidity of some type of adverse event or severe adverse event and its severity increases significantly, researchers must report the adverse event in a timely manner. All severe adverse events must be reported to the drug administration department and the ethics committee within 24 hours (one working day), and the production enterprise must be informed at the same time.

Follow-up

Researchers must follow-up all adverse events and severe adverse events during the trial. Follow-up will continue until the adverse event or the severe adverse event disappears or

For peer review only

For peer review only - http://bmjopen.bmj.com/site/about/guidelines.xhtml

becomes stable. All adverse events are to be kept in the case report form until the last observation date.

Quality control

Quality control is defined as “a part of quality management focused on fulfilling quality requirements” (ISO 9000:2005, Clause 3.2.10). This approach places an emphasis on three aspects: (1) Elements: such as controls, job management, defined and well-managed processes [22], performance and integrity criteria, and identification of records; (2) Competence: such as knowledge, skills, experience, and qualifications; (3) Soft resources: such as personnel, integrity, confidence, organizational culture, motivation, team spirit, and quality relationships. In study management, quality control requires that the project manager and the team inspect the work to ensure its alignment with the project scope [23].

An independent data safety monitoring committee (consist of principal investigators, chief doctors of each centre) has been established to oversee the safety of the trial participants and may suggest terminating the study when the outcome of the interim analysis reaches the determined threshold. Principal investigators will centralize all the data monthly and send a newsletter (a newsletter reports inclusion cases and completed cases of each centre) to participating centre to promote data quality and process of the trial.

Study inspection

For peer review only - http://bmjopen.bmj.com/site/about/guidelines.xhtml

Authorised and qualified researchers will visit the research centres to verify adherence to the protocol and regulations, ensure original data, and to assist research activities according to the inspection plan.

Ethics and dissemination

The protocol has been registered at the ClinicalTrials.gov registry (Protocol ID: HC-I-H 1503; ClinicalTrials.gov ID: NCT02645682.). Any revisions to the protocol will be documented in the ClinicalTrials.gov registry. Written informed consent will be obtained from all participants. All the inclusion patients will be able to have access and correct the data. In case of additional studies from database, all the investigators should keep the results confidential until these are publicly available, and they couldn't give publication related to database without the approval of the principle investigator. We will publish the results of this trial in peer-reviewed clinical journals and present the findings at conferences for widespread dissemination of the results.

Author independence

The study authors affiliated with the West China Hospital and the Peking Union Medical College Hospital designed the study. The authors have full independence in decisions regarding the reporting of results and the content of the reported study.

Trial status

For peer review only - http://bmjopen.bmj.com/site/about/guidelines.xhtml

Data collection is ongoing.

For peer review only

References

1. O'Grady N, Alexander M, Burns LA, et al. Guidelines for the prevention of intravascular catheter-related infections, 2011. Am J Infect Control 2011; 39(4 Suppl 1): S1-34

2. Cicalini S, Palmieri F, Petrosillo N. Clinical review: New technologies for prevention of intravascular catheter-related infections. Crit Care 2004; 8: 157-62

3. Saint S, Veenstra DL, Lipsky BA. The clinical and economic consequences of nosocomial central venous catheter-related infection: are antimicrobial catheters useful? Infection Control and Hospital Epidemiology 2000; 21: 375-80

4. Rupp ME. Central venous catheters coated or impregnated with antimicrobial agents effectively prevent microbial colonisation and catheter-related bloodstream infections. Evid Based Med. 2014; 19: 56

5. Halton KA, Cook DA, Whitby M, et al. Cost effectiveness of antimicrobial catheters in the intensive care unit: addressing uncertainty in the decision. Crit Care 2009; 13: R35

6. Lai NM, Chaiyakunapruk N, Lai NA. Catheter impregnation, coating or bonding for reducing central venous catheter-related infections in adults. Cochrane Database Syst Rev 2013; 6: CD007878

7. Maki DG, Stoltz SM, Wheeler S, et al. Prevention of central venous catheter-related bloodstream infection by use of an antiseptic-impregnated catheter: A randomized, controlled trial. Ann Intern Med1997; 127: 257-66

For peer review only - http://bmjopen.bmj.com/site/about/guidelines.xhtml

8. Raad I, Darouiche R, Dupuis J, et al. Central venous catheters coated with minocycline and rifampin for the prevention of catheter-related colonization and bloodstream infections: A randomized, double-blind trial. Ann Intern Med 1997; 127: 267-74

9. Rupp ME, Lisco SJ, Lipsett PA, et al. Effect of a second generation venous catheter impregnated with chlorhexidine and sliver sulfadiazine on central catheter-related infections: A randomized, controlled trial. Ann Intern Med 2005; 143: 570-80

10. Gong P, Li H, He X, et al. Preparation and antibacterial activity of Fe_3O_4 and Ag nanoparticles. Nanotechnology 2007; 18: 604-11

11. Shrivastava S, Bera T, Roy A, et al. Characterization of enhanced antibacterial effects of novel silver nanoparticles. Nanotechnology 2007; 18: 225103-225112

12. Samuel U, Guggenbichler JP. Prevention of catheter-related infections: the potential of a new nano-silver impregnated catheter. Int J Antimicrob Agents 2004; 23: 75-8

13. Hsu SH, Tseng HJ, Lin YC. The biocompatibility and antibacterial properties of waterborne polyurethane-silver nanocomposites. Biomaterials 2010; 31: 6796-808

14. Mehall JR, Salzman DA, Jackson JR, et al. Fibrin sheath enhances central venous catheter infection. Crit Care Med 2002; 30: 908-12

15. Campisi C, Biffi R, Pittiruti M. Catheter-related venous thrombosis: the development of a nationwide consensus paper in Italy. J AssocVasc Access 2007; 12: 38-46

16. Cortelezzi A, Moia M, Falanga A. Incidence of thrombotic complications in patients with haematological malignancies with central venous catheters: a prospective

For peer review only - http://bmjopen.bmj.com/site/about/guidelines.xhtml

multicenter study. Br J Haematol 2005; 129: 811-7

17. Monreal M, Raventos A, Lerma R, et al. Pulmonary embolism in patients with upper extremity DVT associated to venous central lines–a prospective study. ThrombHaemost1994; 72: 548-50

18. Kearon C, Akl EA, Comerota AJ, et al. Antithrombotic therapy for VTE disease: Antithrombotic Therapy and Prevention of Thrombosis. American College of Chest Physicians Evidence-Based Clinical Practice Guidelines. 9th Ed. Chest2012; 141(Suppl 2): e419S–94S

19. Raad I, Khalil S, Coserton JW, et al. The relationship between the thrombotic and infectious complications of central venous catheters. JAMA 1994; 271: 1014-6

20. Pierce CM, Wade A, Mok Q, et al. Heparin-bonded central venous lines reduce thrombotic and infective complications in critically ill children. Intensive Care Med 2000; 26: 967-72

21. 中华医学会重症医学分会。血管内导管相关感染的预防与治疗指南。中国实用外科杂志 2008; 28: 413-21

22. Dennis Adsit (November 23, 2007). “What the Call Center Industry Can Learn from Manufacturing: Part II” (PDF). National Association of Call Centers. Retrieved 21 December 2012.

23. Phillips, Joseph (November 2008). “Quality Control in Project Management”. The Project Management Hut. Retrieved 21 December 2012.

For peer review only - http://bmjopen.bmj.com/site/about/guidelines.xhtml

Supplemental Appendix

This appendix has been provided by authors to give readers additional information about the research.

For peer review only

Study protocol for a multi-centre, randomized, controlled trial to assess the effectiveness of antimicrobial central venous catheters versus ordinary central venous catheters at reducing catheter-related infections in critically ill Chinese patients

Minming Wu[1], Yao Chen[1], Du Bin[2], Kang Yan[1]

[1] Department of Critical Care Medicine, West China Hospital of Medicine, Sichuan University, No. 37 Guo Xue Xiang, Chengdu, Sichuan, 610041, P.R. China

[2] Medical Intensive Care Unit, Peking Union Medical College Hospital, Peking Union Medical College and Chinese Academy of Medical Sciences, Beijing 100730, China

Corresponding author

Dr Kang Yan

Intensive Care Unit, West China Hospital of Medicine, Sichuan University, Chengdu, Sichuan, 610041, China

Tel.: +86 18980601566

Fax: 85422508

Email: kang_yan_123@163.com

For peer review only

Table of contents

1) Supplemental Background – Study catheter

2) Supplemental Method

2.1) Procedures for insertion

2.2) Care of the catheter during indwelling catheterization

2.3) Remove catheter

2.4) Indication of removal

2.5) Test

2.6) Analysis set

3) Supplemental tables

4）Supplementary Appendix References

For peer review only

1) **Study catheter**

Intervention catheters are antimicrobially modified. Polarization of the catheter material destroys the cell wall structure of microorganisms in case of surface colonization. Perpetual chemical interaction between the polyurethane of the catheter and the agent biguanide ensures the reduction of catheter-related infections during the entire application of the catheter. Control catheters are standard common dual-lumen catheters. The two kinds of catheters are distinguishable in appearance and packaging.

The new antimicrobial CVC (Certofix® protect) was developed by B.Braun to reduce the risk of CRI and CRT. A prospective, randomized, double-bind clinic trial (NCT00555282) conducted in the Czech Republic found that the rate of blood stream infection (BSI) was significantly lower in protected CVCs (2.00 % vs. 6.47 %, p=0.008), and the incidence of BSI/ 1000 catheter-days was lower in coated catheters (3.21 vs. 8.30, p=0.036) as well, but the coated CVC displayed similar incidence of the standard CVCs (17.36 % vs. 18.67 %, p=0.747) as well as incidence of catheter-related BSI (1.33 % vs. 1.94 %, p=0.752) [1].

2) **Supplemental Methods**

Training before trail [2, 3, 4, 5]

2.1) Procedures for insertion

First doctors chose a proper insertion site, and then used maximal barrier precautions during insertion (the operator was required to wear masks, sterile gloves, and surgical gowns and use large sterile drapes). After disinfected with povidone iodine or chlorhexidine, the catheter was inserted percutaneously using Seldinger technique. It was not allowed to exchange the catheter over a guidewire into an old site. Sites were dressed with hyalo-dressing.

2.2) Care of the catheter during indwelling catheterization

Twice a week or according to routine procedures, perform the follows: the dressing removed; the site inspected and cleaned with povidone-iodine or chlorhexidine; and the new dressing applied.

2.3) Remove catheters

At removal, the site was again disinfected by povidone iodine or chlorhexidine to make sure that the skin around the catheter was clean.

For peer review only

2.4) Indication of removal

No need for CVC in patients; Occlusion of catheter; Suspected or confirmed deep vein thrombosis of insertion site; Patients with highly suspected CRBSI and meeting one of the following criteria, haemodynamic instability, bacteremia; or the doctor in charge insisting to remove the catheter after 5 days' observation.

2.5) Tests

Blood culture

For the dual-lumen catheter, blood samples were taken from both lumens separately. Researchers should insert percutaneously to take sample from peripheral blood vessels. Aerobic culture and anaerobic culture were needed for each blood sample.

Cultures of CVC-tip

The entire catheter was removed aseptically, and 4-cm segment was cut from the catheter tip, which was semi-quantitatively cultured using the roll-plate method.

Vein ultrasound

It is used to diagnose or to exclude deep vein thrombosis (DVT). If the insertion site is femoral vein, doctors will screen iliac vein and femoral vein on both sides for DVT. While in the jugular vein, bilateral jugular veins should be inspected. Ultrasound is needless only in case of catheterization in subclavian vein. Ultrasound will be arranged before insertion and after withdrawal of catheter (within 48h).

2.6) Analysis set

Full analysis set (FAS)

The basic intention-to-treat (ITT) principle is that participants in the trials should be analyzed in the groups to which they are randomized, regardless of whether they receive or adhere to the allocated intervention. Based on ITT principle, FAS represents remaining participants after eliminating the least number of patients with reasonable way, including all the patients who are randomized and receive study catheters.

Per protocol set (PPS)

PPS can only be restricted to the participants who fulfill the protocol in the terms of the eligibility, interventions, and outcome assessment. Also, the PPS restricts the comparison of the treatments to the ideal patients, that is, those who adhere perfectly to the clinical trial instructions as stipulated in the protocol. [6]

For peer review only

3) **Supplemental tables**

Table 1 Time to visit and data collection

	Enrollment	Allocation	Post allocation	Closeout
Informed consent	×			
Inclusion/exclusion criteria	×			
Randomization	×			
Medical history & physical examination	×			
Temperature		×	×	
Insertion		×		
Blood test		×	×	
Blood culture			×	
Culture of CVC			×	
Vein ultrasound		×	×	
AE/SAE	×	×	×	×
Treatment/drug combination	×	×	×	×

Table 2 Alpha spending function and cut off value

	Lower bound	Upper bound	Alpha size of test	Alpha spending	Cumulative alpha	Power of test	Overall efficiency
Interim analysis	-2.96259	2.96259	0.003051	0.003051	0.003051	0.164276	0.164276
Final analysis	-1.96857	1.96857	0.049002	0.046949	0.050000	0.636018	0.800294

The distribution of suspension boundary (alpha) is normal distribution.

4) **Supplementary Appendix References**

For peer review only

For peer review only - http://bmjopen.bmj.com/site/about/guidelines.xhtml

1. http://braunoviny.bbraun.cz/clanky/polyhexanide-anti-infective-coating-ofcentral-venous/

2. http://www.safeinfusiontherapy.com/documents/french/Certofix_Brochure.pdf

3. 中华医学会重症医学分会。血管内导管相关感染的预防与治疗指南。中国实用外科杂志 2008; 28: 413-21

4. McGee DC, Gould MK. Preventing complications of central venous catheterization. N Engl J Med 2003;348(12):1123-33.

5. O'Grady N, Alexander M, Burns LA, et al. Guidelines for the prevention of intravascular catheter-related infections, 2011. Am J Infect Control 2011; 39(4 Suppl 1): S1-34

6. U.S. Department of Health and Human Services. Guidance for Industry E9, Statistical Principles for Clinical Trials. 1998.

For peer review only

SPIRIT 2013 Checklist: Recommended items to address in a clinical trial protocol and related documents*

Section/item	Item No	Description	Page No
**Administrative information**			
Title	1	Descriptive title identifying the study design, population, interventions, and, if applicable, trial acronym	1
Trial registration	2a	Trial identifier and registry name. If not yet registered, name of intended registry	4
	2b	All items from the World Health Organization Trial Registration Data Set	4
Protocol version	3	Date and version identifier	4
Funding	4	Sources and types of financial, material, and other support	2
Roles and responsibilities	5a	Names, affiliations, and roles of protocol contributors	2
	5b	Name and contact information for the trial sponsor	1
	5c	Role of study sponsor and funders, if any, in study design; collection, management, analysis, and interpretation of data; writing of the report; and the decision to submit the report for publication, including whether they will have ultimate authority over any of these activities	2
	5d	Composition, roles, and responsibilities of the coordinating centre, steering committee, endpoint adjudication committee, data management team, and other individuals or groups overseeing the trial, if applicable (see Item 21a for data monitoring committee)	17
**Introduction**			
Background and rationale	6a	Description of research question and justification for undertaking the trial, including summary of relevant studies (published and unpublished) examining benefits and harms for each intervention	6
	6b	Explanation for choice of comparators	6
Objectives	7	Specific objectives or hypotheses	6
Trial design	8	Description of trial design including type of trial (eg, parallel group, crossover, factorial, single group), allocation ratio, and framework (eg, superiority, equivalence, noninferiority, exploratory)	6, 9

For peer review only

Methods: Participants, interventions, and outcomes

Study setting	9	Description of study settings (eg, community clinic, academic hospital) and list of countries where data will be collected. Reference to where list of study sites can be obtained	6, 7
Eligibility criteria	10	Inclusion and exclusion criteria for participants. If applicable, eligibility criteria for study centres and individuals who will perform the interventions (eg, surgeons, psychotherapists)	7, 8
Interventions	11a	Interventions for each group with sufficient detail to allow replication, including how and when they will be administered	7, 8, 9
	11b	Criteria for discontinuing or modifying allocated interventions for a given trial participant (eg, drug dose change in response to harms, participant request, or improving/worsening disease)	10
	11c	Strategies to improve adherence to intervention protocols, and any procedures for monitoring adherence (eg, drug tablet return, laboratory tests)	10, 18
	11d	Relevant concomitant care and interventions that are permitted or prohibited during the trial	18
Outcomes	12	Primary, secondary, and other outcomes, including the specific measurement variable (eg, systolic blood pressure), analysis metric (eg, change from baseline, final value, time to event), method of aggregation (eg, median, proportion), and time point for each outcome. Explanation of the clinical relevance of chosen efficacy and harm outcomes is strongly recommended	8
Participant timeline	13	Time schedule of enrolment, interventions (including any run-ins and washouts), assessments, and visits for participants. A schematic diagram is highly recommended (see Figure)	7
Sample size	14	Estimated number of participants needed to achieve study objectives and how it was determined, including clinical and statistical assumptions supporting any sample size calculations	9
Recruitment	15	Strategies for achieving adequate participant enrolment to reach target sample size	9

Methods: Assignment of interventions (for controlled trials)

Allocation:

Sequence generation	16a	Method of generating the allocation sequence (eg, computer-generated random numbers), and list of any factors for stratification. To reduce predictability of a random sequence, details of any planned restriction (eg, blocking) should be provided in a separate document that is unavailable to those who enrol participants or assign interventions	9

Allocation concealment mechanism	16b	Mechanism of implementing the allocation sequence (eg, central telephone; sequentially numbered, opaque, sealed envelopes), describing any steps to conceal the sequence until interventions are assigned	9
Implementation	16c	Who will generate the allocation sequence, who will enrol participants, and who will assign participants to interventions	9
Blinding (masking)	17a	Who will be blinded after assignment to interventions (eg, trial participants, care providers, outcome assessors, data analysts), and how	None
	17b	If blinded, circumstances under which unblinding is permissible, and procedure for revealing a participant's allocated intervention during the trial	None
**Methods: Data collection, management, and analysis**			
Data collection methods	18a	Plans for assessment and collection of outcome, baseline, and other trial data, including any related processes to promote data quality (eg, duplicate measurements, training of assessors) and a description of study instruments (eg, questionnaires, laboratory tests) along with their reliability and validity, if known. Reference to where data collection forms can be found, if not in the protocol	10, 11,17, 18
	18b	Plans to promote participant retention and complete follow-up, including list of any outcome data to be collected for participants who discontinue or deviate from intervention protocols	11, 17
Data management	19	Plans for data entry, coding, security, and storage, including any related processes to promote data quality (eg, double data entry; range checks for data values). Reference to where details of data management procedures can be found, if not in the protocol	10
Statistical methods	20a	Statistical methods for analysing primary and secondary outcomes. Reference to where other details of the statistical analysis plan can be found, if not in the protocol	13
	20b	Methods for any additional analyses (eg, subgroup and adjusted analyses)	14
	20c	Definition of analysis population relating to protocol non-adherence (eg, as randomised analysis), and any statistical methods to handle missing data (eg, multiple imputation)	14
**Methods: Monitoring**			
Data monitoring	21a	Composition of data monitoring committee (DMC); summary of its role and reporting structure; statement of whether it is independent from the sponsor and competing interests; and reference to where further details about its charter can be found, if not in the protocol. Alternatively, an explanation of why a DMC is not needed	12

	21b	Description of any interim analyses and stopping guidelines, including who will have access to these interim results and make the final decision to terminate the trial	12
Harms	22	Plans for collecting, assessing, reporting, and managing solicited and spontaneously reported adverse events and other unintended effects of trial interventions or trial conduct	15, 16
Auditing	23	Frequency and procedures for auditing trial conduct, if any, and whether the process will be independent from investigators and the sponsor	11
**Ethics and dissemination**			
Research ethics approval	24	Plans for seeking research ethics committee/institutional review board (REC/IRB) approval	18
Protocol amendments	25	Plans for communicating important protocol modifications (eg, changes to eligibility criteria, outcomes, analyses) to relevant parties (eg, investigators, REC/IRBs, trial participants, trial registries, journals, regulators)	18
Consent or assent	26a	Who will obtain informed consent or assent from potential trial participants or authorised surrogates, and how (see Item 32)	10, 18
	26b	Additional consent provisions for collection and use of participant data and biological specimens in ancillary studies, if applicable	18
Confidentiality	27	How personal information about potential and enrolled participants will be collected, shared, and maintained in order to protect confidentiality before, during, and after the trial	18
Declaration of interests	28	Financial and other competing interests for principal investigators for the overall trial and each study site	2
Access to data	29	Statement of who will have access to the final trial dataset, and disclosure of contractual agreements that limit such access for investigators	4
Ancillary and post-trial care	30	Provisions, if any, for ancillary and post-trial care, and for compensation to those who suffer harm from trial participation	17
Dissemination policy	31a	Plans for investigators and sponsor to communicate trial results to participants, healthcare professionals, the public, and other relevant groups (eg, via publication, reporting in results databases, or other data sharing arrangements), including any publication restrictions	18
	31b	Authorship eligibility guidelines and any intended use of professional writers	18
	31c	Plans, if any, for granting public access to the full protocol, participant-level dataset, and statistical code	18

For peer review only

Appendices

Informed consent materials	32	Model consent form and other related documentation given to participants and authorised surrogates	Supplemental f
Biological specimens	33	Plans for collection, laboratory evaluation, and storage of biological specimens for genetic or molecular analysis in the current trial and for future use in ancillary studies, if applicable	None

*It is strongly recommended that this checklist be read in conjunction with the SPIRIT 2013 Explanation & Elaboration for important clarification on the items. Amendments to the protocol should be tracked and dated. The SPIRIT checklist is copyrighted by the SPIRIT Group under the Creative Commons "Attribution-NonCommercial-NoDerivs 3.0 Unported" license.

For peer review only

BMJ Open

Study protocol for a multi-centre, randomized, controlled trial to assess the effectiveness of antimicrobial central venous catheters versus ordinary central venous catheters at reducing catheter-related infections in critically ill Chinese patients

Journal:	*BMJ Open*
Manuscript ID	bmjopen-2017-016564.R2
Article Type:	Protocol
Date Submitted by the Author:	21-Oct-2017

Complete List of Authors:	Wu, Minming; Sichuan University West China Hospital, Medical ICU<br>Chen, Yao; Sichuan University West China Hospital, Medical ICU<br>Du, Bin; Peking Union Medical College Hospital, Medical ICU<br>Kang, Yan; Sichuan University West China Hospital, Medical ICU

**Primary Subject Heading**:	Intensive care
Secondary Subject Heading:	Intensive care, Medical management
Keywords:	catheter related infection, central venous catheterization, multi-center randomized controlled trial

SCHOLARONE™ Manuscripts

For peer review only

Study protocol for a multi-centre, randomized, controlled trial to assess the effectiveness of antimicrobial central venous catheters versus ordinary central venous catheters at reducing catheter-related infections in critically ill Chinese patients

Minming Wu[1], Yao Chen[1], Du Bin[2], Kang Yan[1]

[1] Department of Critical Care Medicine, West China Hospital of Medicine, Sichuan University, No. 37 Guo Xue Xiang, Chengdu, Sichuan, 610041, P.R. China

[2] Medical Intensive Care Unit, Peking Union Medical College Hospital, Peking Union Medical College and Chinese Academy of Medical Sciences, Beijing 100730, China

Corresponding author

Dr Kang Yan

Intensive Care Unit, West China Hospital of Medicine, Sichuan University, Chengdu, Sichuan, 610041, China

Tel.: +86 18980601566

Fax: 85422508

Email: kang_yan_123@163.com

For peer review only - http://bmjopen.bmj.com/site/about/guidelines.xhtml

Keywords: central venous catheterization, catheter-related infection, multi-centre randomized controlled trial

Authors' contributions

Prof Du Bin and Prof Kang Yan together designed the study. Dr Minming Wu and Prof Du Bin drafted the manuscript. Dr Kang Yan and Dr Yao Chen critically revised the manuscript. Dr Minming Wu and Dr Yao Chen contributed to the study design and development.

Competing interests

The authors declare that they have no competing financial interests.

Funding

This work is supported by B. Braun Melsungen AG (Melsungen, Germany) through individual research contracts with participating institutions. The funding source has no role in the design or conduct of the trial, data collection, analyses, or manuscript preparation.

Acknowledgments

For peer review only - http://bmjopen.bmj.com/site/about/guidelines.xhtml

This manuscript has been revised by Edanz Editing.

Word count: 2614

For peer review only

For peer review only - http://bmjopen.bmj.com/site/about/guidelines.xhtml

Abstract

Introduction: Catheter use is associated with many complications and is an iatrogenic source of morbidity and mortality in intensive care units (ICU). The catheter being studied (Certofix® protect) was developed to reduce the risk for catheter-related infections. This clinical trial will compare the safety and efficiency of Certofix® protect with that of an ordinary Certofix® catheter.

Methods and analysis: In this multicenter trial, we randomly assigned dual lumen central venous catheterization (≥5ds) in patients in the adult ICU to the antimicrobial CVC group or ordinary CVC group. We planed to recruit 12 to 16 medical centers in China. Our main objective was to assess the effectiveness of antimicrobial CVCs in reducing CRBSI, all-cause mortality, catheter colonization, CRT and other catheter related complications. The primary outcome was the incidence of catheter-related bloodstream infection (CRBSI).

Ethics and dissemination: The Ethics Committee of West China Hospital of Sichuan University has granted ethical approval of this study (27 January 2015). Results will be published in peer-reviewed journals and presented at conferences.

Trial registration: Protocol ID: HC-I-H 1503; ClinicalTrials.gov ID: NCT02645682.

Strengths and limitations of this study

- We include large samples from 12-16 medical centres across different provinces which make results better represent of Chinese ICU patients.

For peer review only

- Our follow-up time is not fixed. The patient will be followed until discharged from the hospital. We may unable to observe the effect of central venous catheterization on long-term quality of life.
- Different puncture skill may influence the risk of mechanical and infectious complications. While our study didn't collect this part of data.

For peer review only

For peer review only - http://bmjopen.bmj.com/site/about/guidelines.xhtml

Introduction

Over the past 30 years, central venous catheters (CVC) have been an essential part of the management of critically and chronically ill patients. However, CVC are associated with a variety of complications including mechanical injury, infection, and thrombosis, and can lead to increased hospital costs and longer hospital stays and mortality [1, 2, 3].

Catheter-related bloodstream infection (CRBSI) is one of the most common, lethal, and costly complications in patients with indwelling CVC [4]. Studies have reported that CVC coated or impregnated with antimicrobial agents could reduce CRBSI and catheter colonization, but didn't reduce systemic infections and all-cause mortality [5 - 13]. Catheter-related thrombosis (CRT) is another common complication of long-term indwelling CVC [14 - 18]. CRT can cause complications such as pulmonary embolism and infection. Critically ill patients with CRBSI are more likely to get CRT [19, 20]. Although many studies on antimicrobial catheters, CRT and relationship between them have been conducted, research in China is limited.

We conducted this multi-centre study to assess the effectiveness of Certofix® protect (supplemental appendix – study catheter) at reducing CRBSI, catheter colonization and CRT in critically ill Chinese adult patients. We also try to find out the relationship between catheter-related infections and CRT.

Methods

For peer review only - http://bmjopen.bmj.com/site/about/guidelines.xhtml

For peer review only

Study design

This is a prospective, multi-centre, parallel group, controlled, randomized clinical trial conducted at 12-16 hospital centres of China from April 2016 until December 2017. The Ethics Committee of West China Hospital of Sichuan University has granted ethical approval of this study (27 January 2015).

Eligibility criteria

Inclusion criteria are: (1) adult patients (>18 years) admitted to an intensive care unit; (2) dual-lumen CVC; (3) patients expected to require indwelling catheterization for at least 5 days; and (4) patients who provide signed informed consent. Peripherally inserted venous catheters, peripherally inserted arterial catheters (including FloTrac®), femoral arterial catheters (including PiCCO®), haemodialysis, pulmonary arterial catheters, and peripherally inserted central catheters can be used in the study. Except for the catheters mentioned above, any other catheter is not permitted.

Exclusion criteria are: (1) pregnant women or women who have recently given birth; (2) patients with malignant diseases and unlikely to survive for the next 28 days in the opinion of the intensive care unit consultant; (3) patients with suspected catheter-related infections; (4) patients receiving an initial study catheter through guidewire exchange; (5) patients hospitalised for severe burn injuries; (6) patients with, in the opinion of the doctors, a situation that is not suitable for indwelling, including allergy to the material of the catheter, confirmed deep vein thrombosis, chronic inflammatory skin disorders at the catheter insertion site, coagulation dysfunction (such as antithrombotic prophylaxis), abnormal anatomical

For peer review only - http://bmjopen.bmj.com/site/about/guidelines.xhtml

structure (enlargement of thyroid glands, cervical tumours, severe pneumonectasis, post-surgical changes of the insertion site); (7) patients who have been enrolled in the study before (during hospitalization); and (8) patients enrolled in another investigative trial in the past 3 months.

The intervention group is those patients that undergo catheterization with Certofix® protect. The control group is those patients that undergo catheterization with Certofix®. Patients are prospectively followed from the day of CVC insertion for at least 5 days or up until CVC removal, whichever comes first. Table 1 shows a schedule for participant enrolment, interventions, assessments, and visits. During treatment, local investigators are required to collect data and samples from patients and arrange tests. All notices are provided in Supplemental appendix.

For peer review only

Table 1 Time to visit and data collection

	Enrollment	Allocation	Post allocation	Closeout
Informed consent	×			
Inclusion/exclusion criteria	×			
Randomization	×			
Medical history & physical examination	×			
Temperature		×	×	
Insertion		×		
Blood test		×	×	
Blood culture			×	

For peer review only - http://bmjopen.bmj.com/site/about/guidelines.xhtml

Culture of CVC			×	
Vein ultrasound		×	×	
AE/SAE	×	×	×	×
Treatment/drug combination	×	×	×	×

Study endpoints

The primary endpoint is CRBSI. CRBSI [21] is defined as CVC-tip colonization by quantitative or semi-quantitative method and at least one peripheral blood culture positive (two separate peripheral blood culture in case of skin contaminant) for the same microorganism or differential time to positivity (>120 min from central and peripheral blood culture). Clinicians should make sure the infection cannot be from another identifiable source of infection. Each suspect case should be discussed with chief doctor of medical group and be presented to an independent data safety monitoring committee. The secondary endpoints are catheter colonization; attack rate of CRT (insertion side or contralateral side); morbidity of CRT (insertion side or contralateral side); and hospital mortality. Catheter colonization [21] is defined as any positive semi-quantitative culture of a distal catheter segment using the roll-plate method (Maki method). The detail description of how and when outcome measures are defined in supplemental appendix – supplemental method.

For peer review only

For peer review only - http://bmjopen.bmj.com/site/about/guidelines.xhtml

Study population

The study sample size is calculated on the basis of an expected CRBSI rate of approximately 6% for the control group and 3% for the antiseptic catheter group. Allowing for a 10% dropout rate, 1818 patients are required to yield a study with 80% power at a statistical significance level of 0.05.

Participant selection and recruitment

Before identifying and screening patients for eligibility, informed consent (supplemental file) must be obtained by the doctor in charge. All information is to be transferred into an electronic database so that the trial office can monitor recruitment and refusal rates at each centre.

Randomization

Each research centre will receive sequentially numbered containers used to implement the random allocation sequence. And the treatment allocation group is hiding beyond the coated card. To ensure that patients are randomly assigned at a 1:1 ratio at each study centre, the randomized cards was protected using a block design (each block includes 4 random allocation sequence). For a patient who meets the required criteria, the local investigator opens a randomized card that records the screening number and treatment allocation group. Then, physician in charge of the patient will obtain the right study catheter and complete

For peer review only - http://bmjopen.bmj.com/site/about/guidelines.xhtml

catheterization. So that treatment allocation is concealed.

Patient termination and withdrawal criteria

Participants and their authorised surrogates will participate in the study voluntarily, therefore, they may withdraw from the trial at any time for any reason. Patients may also be withdrawn from the study for: (1) severe adverse events; or (2) violating or deviating from the protocol. If a patient is withdrawn for one of the two reasons mentioned, they should proceed to security analysis.

Research centre termination and withdrawal criteria

A research centre must terminate their involvement in the clinical trial if: (1) the researchers do not obey the rules of the International Conference on Harmonisation Guidelines for Good Clinical Practice or local regulations; (2) the research centre intentionally submits incorrect or incomplete data to inspectors; (3) the requirements of the protocol are not met, including poor data quality (incomplete case report forms); or (4) investigators make changes without informing the lead researchers. Each investigator should be qualified and be approved by the lead researchers. As a 10% dropout rate is allowed, there will be no need to add new patients when an existing participant withdraws from the trial.

Data collection and inspection

For peer review only - http://bmjopen.bmj.com/site/about/guidelines.xhtml

Principal investigators will centralize all the data monthly and send a newsletter to each centre to promote data quality and process of the trial. Data collection begins on the day a participant signs the informed consent and continues until the participant is discharged or transferred to another hospital. Data are collected using a paper-based case report form (supplemental file, data collection form) and an electronic database.

Investigators follow a schedule to collect data, including: (1) screening data, informed consent, demographic data, inclusion and exclusion criteria, and enrolment data; (2) baseline information on catheterization (age, male, ID, height, weight, risk factor of infection, SOFA score, APACH2 score, underlying diseases, antibiotic therapy), vascular ultrasound of veins at the insertion site and contralateral site, and CVC catheterization (date, temperature, catheter type, insertion site, Neutrophil count, antibiotic therapy, other type of catheterization, SAE); (3) CVC removal data (duration of catheterization, temperature, reason for catheter removal, parenteral nutrition, Neutrophil count), peripheral blood cultures, catheter blood cultures, catheter tip cultures, and vascular ultrasound of veins at the insertion site and contralateral site; and (4) prognosis, date of transferring out of the intensive care unit, and date of discharge/death, whichever comes first.

Follow-up data

Statistical analysis plan

Hypothesis

For peer review only - http://bmjopen.bmj.com/site/about/guidelines.xhtml

The study hypothesis is:

H_0: πCVCp = πCVC

H_1: πCVCp < πCVC

Where π represents the incidence of CRBSI.

Analysis sets

There will be a full analysis set, a per protocol set, and a safety set (supplemental appendix – supplemental method).

Statistical analysis

Principles

All statistical tests will be two tailed and will be analysed using SAS statistical analysis software (ver. 9.4; SAS Institute, Cary, NC). Quantitative variables will be analysed by calculating the mean, standard deviation, median, minimum value, maximum value, lower quartile (Q1), and upper quartile (Q3). Categorical variables will be described using cases and percentages for each category. The significance of differences between two groups will be determined using the chi-square test or Fisher's exact test for categorical data, the group *t*-test or Wilcoxon rank sum test for continuous data, and the Wilcoxon rank sum test or the Cochran–Mantel–Haenszel chi-square test for ranked data.

For peer review only - http://bmjopen.bmj.com/site/about/guidelines.xhtml

For peer review only

Proposed primary analysis

The incidence density of CRBSI in the two groups will be compared using the Cochran–Mantel–Haenszel chi-square test and stratified analysis based on the time CRBSI occurs. For the interim analysis, the size of the test for α_1 is 0.003, and we will also calculate $(1-\alpha_1) \times 100\%$ confidence intervals. If the result rejects H_0, then the antimicrobial CVC group is superior to the ordinary CVC group. If the interim analysis shows no statistical significance or if the data safety monitoring board decides to complete the next stage of the trial, then we will complete the final analysis ($\alpha_2 = 0.049$, CI $(1-\alpha_2) \times 100\%$). The proposed primary analysis is based on the final analysis set and the per protocol set. Table 2 shows the alpha spending functions and cut-off values.

Table 2 Alpha spending function and cut off value

	Lower bound	Upper bound	Alpha size of test	Alpha spending	Cumulative alpha	Power of test	Overall efficiency
Interim analysis	-2.96259	2.96259	0.003051	0.003051	0.003051	0.164276	0.164276
Final analysis	-1.96857	1.96857	0.049002	0.046949	0.050000	0.636018	0.800294

The distribution of suspension boundary (alpha) is normal distribution.

Secondary analysis

Incidence density of Catheter-tip colonization, CRT, and hospital mortality in the two groups

For peer review only - http://bmjopen.bmj.com/site/about/guidelines.xhtml

will be compared using the chi-square test or Fisher's exact test, or random-intercept logistic regression. Analyses of the other indicators follows the process described under "Principles" above. Analyses of the secondary indicators is based on the full analysis set and the per protocol set.

Subgroup analysis

Subgroup analyses will be conducted for predefined factors such as insertion site, catheter durations, antibiotic therapy, anticoagulation therapy, underlying diseases, BMI, SOFA score, APACHE2 score, etc. Other exploratory subgroup analyses will be eventually conducted.

Safety analysis

The proportion of abnormal cases after treatment will be determined, as will the number of cases/incidence of adverse events and severe adverse events. We will also describe the clinical manifestations, degrees of all adverse events, and the relationship between these factors and the catheters in detail. Changes in indexes will be described using a crosstab grid. All safety evaluations will be based on the safety set.

Missing data

For peer review only - http://bmjopen.bmj.com/site/about/guidelines.xhtml

Worsts Observation Carried Forward will be used to evaluate missing data in the full analysis set. Dropout rates will be obtained and for each group we will determine if the dropout rate is higher than the difference in event rates between the two groups using the worst-case scenario model.

Proposed interim analysis

An interim analysis will be conducted in the middle of the recruitment period to evaluate the effectiveness of the main indexes and to determine whether it is necessary/possible to terminate the trial early.

Adverse events

Definitions

An adverse event is defined as a patient who develops clinical features such as discomfort or laboratory abnormalities that are not related to the expected therapeutic effects during central venous catheterization.

The catheter-associated adverse events according to the modified CTCAE V.4 classification [22] to be recorded are: (1) a broken or cracked catheter; (2) hematoma at the insertion site; (3) chylothorax, pneumothorax, haemothorax, or pleural effusion caused by mispuncture or

For peer review only - http://bmjopen.bmj.com/site/about/guidelines.xhtml

malposition; (4) arrhythmia or rupture of the atrium caused by malposition, endocarditis because of mechanical stimulation, thrombophlebitis, or injury to the atrium, thoracic duct, brachial plexus, or phrenic nerve because of mispuncture.

The severe adverse events (definitely related or possibly related) to be recorded are: (1) death as the result of an adverse event. Medical conditions resulting in death need to be comprehensively reported, such as an underlying disease or an accident; (2) life-threatening events. Life-threatening events are those events that put the patient at risk for death at the time. This is distinct from an event that may become more serious in the future and put the patient at risk for death; (3) events requiring hospitalization or prolong the time of hospitalization. Hospitalization in this context means more than 1 calendar day; (4) events leading to permanent damage, or medical intervention that must be taken to avoid permanent damage.

An event may meet more than one criteria. If the event could result in harm to a patient or clinician, intervention should be taken to prevent the event, and this adverse event should be recorded as a severe adverse event.

Recording and reporting

Researchers must record adverse events and severe adverse events in the corresponding case report form, including signs and symptoms, date, disappearance date (duration), severity or strength, relationship with therapy, measurements, and outcomes. If the interim analysis finds that the morbidity of some type of adverse event or severe adverse event and its severity

For peer review only

increases significantly, researchers must report the adverse event in a timely manner. All severe adverse events must be reported to the drug administration department and the ethics committee within 24 hours (one working day), and the production enterprise must be informed at the same time.

Follow-up

Researchers must follow-up all adverse events and severe adverse events during the trial. Follow-up will continue until the adverse event or the severe adverse event disappears or becomes stable. All adverse events are to be kept in the case report form until the last observation date.

Quality control

Quality control is defined as "a part of quality management focused on fulfilling quality requirements" (ISO 9000:2005, Clause 3.2.10). This approach places an emphasis on three aspects: (1) Elements: such as controls, job management, defined and well-managed processes [23], performance and integrity criteria, and identification of records; (2) Competence: such as knowledge, skills, experience, and qualifications; (3) Soft resources: such as personnel, integrity, confidence, organizational culture, motivation, team spirit, and quality relationships. In study management, quality control requires that the project manager and the team inspect the work to ensure its alignment with the project scope [24].

For peer review only - http://bmjopen.bmj.com/site/about/guidelines.xhtml

An independent data safety monitoring committee (consist of experts of each centre who are not investigators) has been established to oversee the safety of the trial participants and may suggest terminating the study when the outcome of the interim analysis reaches the determined threshold. Principal investigators will centralize all the data monthly and send a newsletter (a newsletter reports inclusion cases and completed cases of each centre) to participating centre to promote data quality and process of the trial.

Study inspection

Authorised and qualified researchers will visit the research centres to verify adherence to the protocol and regulations, ensure original data, and to assist research activities according to the inspection plan.

Ethics and dissemination

The protocol has been registered at the ClinicalTrials.gov registry (Protocol ID: HC-I-H 1503; ClinicalTrials.gov ID: NCT02645682.). Any revisions to the protocol will be documented in the ClinicalTrials.gov registry. Written informed consent will be obtained from all participants. All the inclusion patients will be able to have access and correct the data. In case of additional studies from database, all the investigators should keep the results confidential until these are publicly available, and they couldn’t give publication related to database without the approval of the principle investigator. We will publish the results of this trial in

For peer review only

peer-reviewed clinical journals and present the findings at conferences for widespread dissemination of the results.

Author independence

The study authors affiliated with the West China Hospital and the Peking Union Medical College Hospital designed the study. The authors have full independence in decisions regarding the reporting of results and the content of the reported study.

Trial status

Data collection is ongoing.

For peer review only

For peer review only - http://bmjopen.bmj.com/site/about/guidelines.xhtml

References

1. O'Grady N, Alexander M, Burns LA, et al. Guidelines for the prevention of intravascular catheter-related infections, 2011. Am J Infect Control 2011; 39(4 Suppl 1): S1-34

2. Cicalini S, Palmieri F, Petrosillo N. Clinical review: New technologies for prevention of intravascular catheter-related infections. Crit Care 2004; 8: 157-62

3. Saint S, Veenstra DL, Lipsky BA. The clinical and economic consequences of nosocomial central venous catheter-related infection: are antimicrobial catheters useful? Infection Control and Hospital Epidemiology 2000; 21: 375-80

4. Rupp ME. Central venous catheters coated or impregnated with antimicrobial agents effectively prevent microbial colonisation and catheter-related bloodstream infections. Evid Based Med. 2014; 19: 56

5. Halton KA, Cook DA, Whitby M, et al. Cost effectiveness of antimicrobial catheters in the intensive care unit: addressing uncertainty in the decision. Crit Care 2009; 13: R35

6. Lai NM, Chaiyakunapruk N, Lai NA. Catheter impregnation, coating or bonding for reducing central venous catheter-related infections in adults. Cochrane Database Syst Rev 2013; 6: CD007878

7. Maki DG, Stoltz SM, Wheeler S, et al. Prevention of central venous catheter-related bloodstream infection by use of an antiseptic-impregnated catheter: A randomized, controlled trial. Ann Intern Med1997; 127: 257-66

For peer review only - http://bmjopen.bmj.com/site/about/guidelines.xhtml

8. Raad I, Darouiche R, Dupuis J, et al. Central venous catheters coated with minocycline and rifampin for the prevention of catheter-related colonization and bloodstream infections: A randomized, double-blind trial. Ann Intern Med 1997; 127: 267-74

9. Rupp ME, Lisco SJ, Lipsett PA, et al. Effect of a second generation venous catheter impregnated with chlorhexidine and sliver sulfadiazine on central catheter-related infections: A randomized, controlled trial. Ann Intern Med 2005; 143: 570-80

10. Gong P, Li H, He X, et al. Preparation and antibacterial activity of Fe_3O_4 and Ag nanoparticles. Nanotechnology 2007; 18: 604-11

11. Shrivastava S, Bera T, Roy A, et al. Characterization of enhanced antibacterial effects of novel silver nanoparticles. Nanotechnology 2007; 18: 225103-225112

12. Samuel U, Guggenbichler JP. Prevention of catheter-related infections: the potential of a new nano-silver impregnated catheter. Int J Antimicrob Agents 2004; 23: 75-8

13. Hsu SH, Tseng HJ, Lin YC. The biocompatibility and antibacterial properties of waterborne polyurethane-silver nanocomposites. Biomaterials 2010; 31: 6796-808

14. Mehall JR, Salzman DA, Jackson JR, et al. Fibrin sheath enhances central venous catheter infection. Crit Care Med 2002; 30: 908-12

15. Campisi C, Biffi R, Pittiruti M. Catheter-related venous thrombosis: the development of a nationwide consensus paper in Italy. J AssocVasc Access 2007; 12: 38-46

16. Cortelezzi A, Moia M, Falanga A. Incidence of thrombotic complications in patients with haematological malignancies with central venous catheters: a prospective

For peer review only - http://bmjopen.bmj.com/site/about/guidelines.xhtml

multicenter study. Br J Haematol 2005; 129: 811-7

17. Monreal M, Raventos A, Lerma R, et al. Pulmonary embolism in patients with upper extremity DVT associated to venous central lines–a prospective study. ThrombHaemost1994; 72: 548-50

18. Kearon C, Akl EA, Comerota AJ, et al. Antithrombotic therapy for VTE disease: Antithrombotic Therapy and Prevention of Thrombosis. American College of Chest Physicians Evidence-Based Clinical Practice Guidelines. 9th Ed. Chest2012; 141(Suppl 2): e419S–94S

19. Raad I, Khalil S, Coserton JW, et al. The relationship between the thrombotic and infectious complications of central venous catheters. JAMA 1994; 271: 1014-6

20. Pierce CM, Wade A, Mok Q, et al. Heparin-bonded central venous lines reduce thrombotic and infective complications in critically ill children. Intensive Care Med 2000; 26: 967-72

21. 中华医学会重症医学分会。血管内导管相关感染的预防与治疗指南。中国实用外科杂志 2008; 28: 413-21

22. Parienti, J. J., N. Mongardon, B. Megarbane, J. P. Mira, P. Kalfon, A. Gros, et al. 2015. Intravascular complications of central venous catheterization by insertion site. N. Engl. J. Med. 373:1220–1229

23. Dennis Adsit (November 23, 2007). "What the Call Center Industry Can Learn from Manufacturing: Part II" (PDF). National Association of Call Centers. Retrieved 21

For peer review only - http://bmjopen.bmj.com/site/about/guidelines.xhtml

December 2012.

24. Phillips, Joseph (November 2008). “Quality Control in Project Management”. The Project Management Hut. Retrieved 21 December 2012.

For peer review only

Supplemental Appendix

This appendix has been provided by authors to give readers additional information about the research.

For peer review only

Study protocol for a multi-centre, randomized, controlled trial to assess the effectiveness of antimicrobial central venous catheters versus ordinary central venous catheters at reducing catheter-related infections in critically ill Chinese patients

Minming Wu[1], Yao Chen[1], Du Bin[2], Kang Yan[1]

[1] Department of Critical Care Medicine, West China Hospital of Medicine, Sichuan University, No. 37 Guo Xue Xiang, Chengdu, Sichuan, 610041, P.R. China

[2] Medical Intensive Care Unit, Peking Union Medical College Hospital, Peking Union Medical College and Chinese Academy of Medical Sciences, Beijing 100730, China

Corresponding author

Dr Kang Yan

Intensive Care Unit, West China Hospital of Medicine, Sichuan University, Chengdu, Sichuan, 610041, China

Tel.: +86 18980601566

Fax: 85422508

Email: kang_yan_123@163.com

For peer review only

Table of contents

1) Supplemental Background – Study catheter

2) Supplemental Method

2.1) Procedures for insertion

2.2) Care of the catheter during indwelling catheterization

2.3) Remove catheter

2.4) Indication of removal

2.5) Test

2.6) Analysis set

3) Supplemental tables

4）Supplementary Appendix References

For peer review only

1) **Study catheter**

Intervention catheters are antimicrobially modified. Polarization of the catheter material destroys the cell wall structure of microorganisms in case of surface colonization. Perpetual chemical interaction between the polyurethane of the catheter and the agent biguanide ensures the reduction of catheter-related infections during the entire application of the catheter. Control catheters are standard common dual-lumen catheters. The two kinds of catheters are distinguishable in appearance and packaging.

The new antimicrobial CVC (Certofix® protect) was developed by B.Braun to reduce the risk of CRI and CRT. A prospective, randomized, double-bind clinic trial (NCT00555282) conducted in the Czech Republic found that the rate of blood stream infection (BSI) was significantly lower in protected CVCs (2.00 % vs. 6.47 %, p=0.008), and the incidence of BSI/ 1000 catheter-days was lower in coated catheters (3.21 vs. 8.30, p=0.036) as well, but the coated CVC displayed similar incidence of the standard CVCs (17.36 % vs. 18.67 %, p=0.747) as well as incidence of catheter-related BSI (1.33 % vs. 1.94 %, p=0.752) [1].

2) **Supplemental Methods**

Training before trail [2, 3, 4, 5]

2.1) Procedures for insertion

First doctors chose a proper insertion site, and then used maximal barrier precautions during insertion (the operator was required to wear masks, sterile gloves, and surgical gowns and use large sterile drapes). After disinfected with povidone iodine or chlorhexidine, the catheter was inserted percutaneously using Seldinger technique. It was not allowed to exchange the catheter over a guidewire into an old site. Sites were dressed with hyalo-dressing.

2.2) Care of the catheter during indwelling catheterization

Twice a week or according to routine procedures, perform the follows: the dressing removed; the site inspected and cleaned with povidone-iodine or chlorhexidine; and the new dressing applied.

2.3) Remove catheters

At removal, the site was again disinfected by povidone iodine or chlorhexidine to make sure that the skin around the catheter was clean.

For peer review only

2.4) Indication of removal

No need for CVC in patients; Occlusion of catheter; Suspected or confirmed deep vein thrombosis of insertion site; Patients with highly suspected CRBSI and meeting one of the following criteria, haemodynamic instability, bacteremia; or the doctor in charge insisting to remove the catheter after 5 days' observation.

2.5) Tests

Blood culture

For the dual-lumen catheter, blood samples were taken from both lumens separately. Researchers should insert percutaneously to take sample from peripheral blood vessels. Aerobic culture and anaerobic culture were needed for each blood sample.

Cultures of CVC-tip

The entire catheter was removed aseptically, and 4-cm segment was cut from the catheter tip, which was semi-quantitatively cultured using the roll-plate method.

Vein ultrasound

It is used to diagnose or to exclude deep vein thrombosis (DVT). If the insertion site is femoral vein, doctors will screen iliac vein and femoral vein on both sides for DVT. While in the jugular vein, bilateral jugular veins should be inspected. Ultrasound is needless only in case of catheterization in subclavian vein. Ultrasound will be arranged before insertion and after withdrawal of catheter (within 48h).

2.6) Analysis set

Full analysis set (FAS)

The basic intention-to-treat (ITT) principle is that participants in the trials should be analyzed in the groups to which they are randomized, regardless of whether they receive or adhere to the allocated intervention. Based on ITT principle, FAS represents remaining participants after eliminating the least number of patients with reasonable way, including all the patients who are randomized and receive study catheters.

Per protocol set (PPS)

PPS can only be restricted to the participants who fulfill the protocol in the terms of the eligibility, interventions, and outcome assessment. Also, the PPS restricts the comparison of the treatments to the ideal patients, that is, those who adhere perfectly to the clinical trial instructions as stipulated in the protocol. [6]

For peer review only

3) **Supplemental tables**

Table 1 Time to visit and data collection

	Enrollment	Allocation	Post allocation	Closeout
Informed consent	×			
Inclusion/exclusion criteria	×			
Randomization	×			
Medical history & physical examination	×			
Temperature		×	×	
Insertion		×		
Blood test		×	×	
Blood culture			×	
Culture of CVC			×	
Vein ultrasound		×	×	
AE/SAE	×	×	×	×
Treatment/drug combination	×	×	×	×

For peer review only

Table 2 Alpha spending function and cut off value

	Lower bound	Upper bound	Alpha size of test	Alpha spending	Cumulative alpha	Power of test	Overall efficiency
Interim analysis	-2.96259	2.96259	0.003051	0.003051	0.003051	0.164276	0.164276
Final analysis	-1.96857	1.96857	0.049002	0.046949	0.050000	0.636018	0.800294

The distribution of suspension boundary (alpha) is normal distribution.

4) **Supplementary Appendix References**

1. http://braunoviny.bbraun.cz/clanky/polyhexanide-anti-infective-coating-ofcentral-venous/

2. http://www.safeinfusiontherapy.com/documents/french/Certofix_Brochure.pdf

3. 中华医学会重症医学分会。血管内导管相关感染的预防与治疗指南。中国实用外科杂志 2008; 28: 413-21

4. McGee DC, Gould MK. Preventing complications of central venous catheterization. N Engl J Med 2003;348(12):1123-33.

5. O'Grady N, Alexander M, Burns LA, et al. Guidelines for the prevention of intravascular catheter-related infections, 2011. Am J Infect Control 2011; 39(4 Suppl 1): S1-34

6. U.S. Department of Health and Human Services. Guidance for Industry E9, Statistical Principles for Clinical Trials. 1998.

For peer review only

STANDARD PROTOCOL ITEMS: RECOMMENDATIONS FOR INTERVENTIONAL TRIALS

SPIRIT 2013 Checklist: Recommended items to address in a clinical trial protocol and related documents*

Section/item	Item No	Description	Page No
**Administrative information**			
Title	1	Descriptive title identifying the study design, population, interventions, and, if applicable, trial acronym	1
Trial registration	2a	Trial identifier and registry name. If not yet registered, name of intended registry	4
	2b	All items from the World Health Organization Trial Registration Data Set	4
Protocol version	3	Date and version identifier	4
Funding	4	Sources and types of financial, material, and other support	2
Roles and responsibilities	5a	Names, affiliations, and roles of protocol contributors	2
	5b	Name and contact information for the trial sponsor	1
	5c	Role of study sponsor and funders, if any, in study design; collection, management, analysis, and interpretation of data; writing of the report; and the decision to submit the report for publication, including whether they will have ultimate authority over any of these activities	2
	5d	Composition, roles, and responsibilities of the coordinating centre, steering committee, endpoint adjudication committee, data management team, and other individuals or groups overseeing the trial, if applicable (see Item 21a for data monitoring committee)	17
**Introduction**			
Background and rationale	6a	Description of research question and justification for undertaking the trial, including summary of relevant studies (published and unpublished) examining benefits and harms for each intervention	6
	6b	Explanation for choice of comparators	6
Objectives	7	Specific objectives or hypotheses	6
Trial design	8	Description of trial design including type of trial (eg, parallel group, crossover, factorial, single group), allocation ratio, and framework (eg, superiority, equivalence, noninferiority, exploratory)	6, 9

For peer review only

Methods: Participants, interventions, and outcomes

Study setting	9	Description of study settings (eg, community clinic, academic hospital) and list of countries where data will be collected. Reference to where list of study sites can be obtained	6, 7
Eligibility criteria	10	Inclusion and exclusion criteria for participants. If applicable, eligibility criteria for study centres and individuals who will perform the interventions (eg, surgeons, psychotherapists)	7, 8
Interventions	11a	Interventions for each group with sufficient detail to allow replication, including how and when they will be administered	7, 8, 9
	11b	Criteria for discontinuing or modifying allocated interventions for a given trial participant (eg, drug dose change in response to harms, participant request, or improving/worsening disease)	10
	11c	Strategies to improve adherence to intervention protocols, and any procedures for monitoring adherence (eg, drug tablet return, laboratory tests)	10, 18
	11d	Relevant concomitant care and interventions that are permitted or prohibited during the trial	18
Outcomes	12	Primary, secondary, and other outcomes, including the specific measurement variable (eg, systolic blood pressure), analysis metric (eg, change from baseline, final value, time to event), method of aggregation (eg, median, proportion), and time point for each outcome. Explanation of the clinical relevance of chosen efficacy and harm outcomes is strongly recommended	8
Participant timeline	13	Time schedule of enrolment, interventions (including any run-ins and washouts), assessments, and visits for participants. A schematic diagram is highly recommended (see Figure)	7
Sample size	14	Estimated number of participants needed to achieve study objectives and how it was determined, including clinical and statistical assumptions supporting any sample size calculations	9
Recruitment	15	Strategies for achieving adequate participant enrolment to reach target sample size	9

Methods: Assignment of interventions (for controlled trials)

Allocation:

Sequence generation	16a	Method of generating the allocation sequence (eg, computer-generated random numbers), and list of any factors for stratification. To reduce predictability of a random sequence, details of any planned restriction (eg, blocking) should be provided in a separate document that is unavailable to those who enrol participants or assign interventions	9

Allocation concealment mechanism	16b	Mechanism of implementing the allocation sequence (eg, central telephone; sequentially numbered, opaque, sealed envelopes), describing any steps to conceal the sequence until interventions are assigned	9
Implementation	16c	Who will generate the allocation sequence, who will enrol participants, and who will assign participants to interventions	9
Blinding (masking)	17a	Who will be blinded after assignment to interventions (eg, trial participants, care providers, outcome assessors, data analysts), and how	None
	17b	If blinded, circumstances under which unblinding is permissible, and procedure for revealing a participant's allocated intervention during the trial	None
**Methods: Data collection, management, and analysis**			
Data collection methods	18a	Plans for assessment and collection of outcome, baseline, and other trial data, including any related processes to promote data quality (eg, duplicate measurements, training of assessors) and a description of study instruments (eg, questionnaires, laboratory tests) along with their reliability and validity, if known. Reference to where data collection forms can be found, if not in the protocol	10, 11,17, 18
	18b	Plans to promote participant retention and complete follow-up, including list of any outcome data to be collected for participants who discontinue or deviate from intervention protocols	11, 17
Data management	19	Plans for data entry, coding, security, and storage, including any related processes to promote data quality (eg, double data entry; range checks for data values). Reference to where details of data management procedures can be found, if not in the protocol	10
Statistical methods	20a	Statistical methods for analysing primary and secondary outcomes. Reference to where other details of the statistical analysis plan can be found, if not in the protocol	13
	20b	Methods for any additional analyses (eg, subgroup and adjusted analyses)	14
	20c	Definition of analysis population relating to protocol non-adherence (eg, as randomised analysis), and any statistical methods to handle missing data (eg, multiple imputation)	14
**Methods: Monitoring**			
Data monitoring	21a	Composition of data monitoring committee (DMC); summary of its role and reporting structure; statement of whether it is independent from the sponsor and competing interests; and reference to where further details about its charter can be found, if not in the protocol. Alternatively, an explanation of why a DMC is not needed	12

For peer review only - http://bmjopen.bmj.com/site/about/guidelines.xhtml

	21b	Description of any interim analyses and stopping guidelines, including who will have access to these interim results and make the final decision to terminate the trial	12
Harms	22	Plans for collecting, assessing, reporting, and managing solicited and spontaneously reported adverse events and other unintended effects of trial interventions or trial conduct	15, 16
Auditing	23	Frequency and procedures for auditing trial conduct, if any, and whether the process will be independent from investigators and the sponsor	11
**Ethics and dissemination**			
Research ethics approval	24	Plans for seeking research ethics committee/institutional review board (REC/IRB) approval	18
Protocol amendments	25	Plans for communicating important protocol modifications (eg, changes to eligibility criteria, outcomes, analyses) to relevant parties (eg, investigators, REC/IRBs, trial participants, trial registries, journals, regulators)	18
Consent or assent	26a	Who will obtain informed consent or assent from potential trial participants or authorised surrogates, and how (see Item 32)	10, 18
	26b	Additional consent provisions for collection and use of participant data and biological specimens in ancillary studies, if applicable	18
Confidentiality	27	How personal information about potential and enrolled participants will be collected, shared, and maintained in order to protect confidentiality before, during, and after the trial	18
Declaration of interests	28	Financial and other competing interests for principal investigators for the overall trial and each study site	2
Access to data	29	Statement of who will have access to the final trial dataset, and disclosure of contractual agreements that limit such access for investigators	4
Ancillary and post-trial care	30	Provisions, if any, for ancillary and post-trial care, and for compensation to those who suffer harm from trial participation	17
Dissemination policy	31a	Plans for investigators and sponsor to communicate trial results to participants, healthcare professionals, the public, and other relevant groups (eg, via publication, reporting in results databases, or other data sharing arrangements), including any publication restrictions	18
	31b	Authorship eligibility guidelines and any intended use of professional writers	18
	31c	Plans, if any, for granting public access to the full protocol, participant-level dataset, and statistical code	18

For peer review

Appendices

Informed consent materials	32	Model consent form and other related documentation given to participants and authorised surrogates	Supplemental f
Biological specimens	33	Plans for collection, laboratory evaluation, and storage of biological specimens for genetic or molecular analysis in the current trial and for future use in ancillary studies, if applicable	None

*It is strongly recommended that this checklist be read in conjunction with the SPIRIT 2013 Explanation & Elaboration for important clarification on the items. Amendments to the protocol should be tracked and dated. The SPIRIT checklist is copyrighted by the SPIRIT Group under the Creative Commons "Attribution-NonCommercial-NoDerivs 3.0 Unported" license.

For peer review only
